# A DNP‐Supported Solid‐State NMR Approach to Study Nucleic Acids In Situ Reveals Berberine‐Stabilized Hoogsteen Structures in Mitochondria

**DOI:** 10.1002/anie.202424131

**Published:** 2025-03-18

**Authors:** Michaela Dzurov Krafčíková, David Beriashvili, Salima Bahri, Menno Bergmeijer, Stuart C. Howes, Andrei Gurinov, Friedrich G. Förster, Gert E. Folkers, Marc Baldus

**Affiliations:** ^1^ NMR Spectroscopy Bijvoet Center for Biomolecular Research Utrecht University Padualaan 8 Utrecht 3584CH The Netherlands; ^2^ Structural Biochemistry Bijvoet Center for Biomolecular Research Utrecht University Universiteitsweg 99 Utrecht 3584CG The Netherlands

**Keywords:** Berberine, DNA/RNA, DNP‐ssNMR, G‐quadruplex, Mitochondria

## Abstract

Mitochondria are central to cellular bioenergetics, with the unique ability to translate and transcribe a subset of their own proteome. Given the critical importance of energy production, mitochondria seem to utilize higher‐order nucleic acid structures to regulate gene expression, much like nuclei. Herein, we introduce a tailored approach to probe the formation of such structures, specifically G‐quadruplexes, within intact mitochondria by using sensitivity‐enhanced dynamic nuclear polarization‐supported solid‐state NMR (DNP‐ssNMR). We acquired NMR spectra on isolated intact isotopically labeled mitochondria treated with berberine, a known high‐affinity G‐quadruplex stabilizer. The DNP‐ssNMR data revealed spectral changes in nucleic acid sugar correlations, increased signal intensity for guanosine carbons, and enhanced Hoogsteen hydrogen bond formation, providing evidence of in vivo G‐quadruplex formation in mitochondria. Together, our workflow enables the study of mitochondrial nucleic acid‐ligand interactions at endogenous concentrations within biologically relevant environments by DNP‐ssNMR, thus paving the way for future research into mitochondrial diseases and their potential treatments.

## Graphical abstract

Mitochondria are vital for energy production and rely on intricate nucleic acid structures to regulate gene expression. Using DNP‐ssNMR (dynamic nuclear polarization solid‐state NMR), we observed G‐quadruplex formation in intact mitochondria treated with berberine. This method enables detailed studies of mitochondrial nucleic acids in their native environment, advancing research into mitochondrial diseases and therapies.

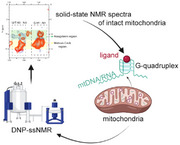

## Introduction

Mitochondria are vital organelles responsible for cellular energy production via oxidative phosphorylation (OXPHOS) and play key roles in metabolic signaling, bioenergetics, calcium transport, reactive oxygen species production, and cell death regulation.^[^ ^1^ ^]^ Dysfunction of these organelles is implicated in diseases such as Leigh syndrome, epilepsy, cardiac disorders, inflammation, and cancer, underscoring the importance of understanding mitochondrial biology.^[^ ^2^ ^]^

A defining feature of mitochondria is their unique genome, which encodes key OXPHOS proteins and their associated translation machinery. Human mitochondrial DNA (mtDNA), a compact 16600 base pair circular genome, encodes 13 proteins, 2 ribosomal RNAs, and 22 transfer RNAs, with minimal noncoding sequences.^[^ ^3^ ^]^ This stands in contrast to the largely noncoding, linear nuclear genome.^[^ ^4^ ^]^ The rest of the mitochondrial proteome is synthesized in the cytosol and imported into mitochondria.

Nucleic acids, such as mtDNA and associated RNAs, are essential for mitochondrial function and broader cellular processes. Studying them in situ—within the organelle's native environment—is crucial for understanding their spatial organization, interactions, and functional dynamics. Experimental evidence suggests that non‐canonical DNA/RNA structures such as G‐quadruplexes (G4s) play crucial roles in regulating mitochondrial transcription and translation,^[^ ^5^, ^6^, ^7^, ^8^ ^]^ much like in the nucleus. Analysis of mitochondrial genomes has revealed that putative quadruplex‐forming sequences (PQS) occur more frequently in key regulatory regions, such as the 3′UTRs, D‐loops, origins of replication, and stem‐loops, thus highlighting the great potential of PQSs as targets in therapeutic intervention.^[^ ^9^, ^10^, ^11^, ^12^, ^13^ ^]^

**Figure 1:**
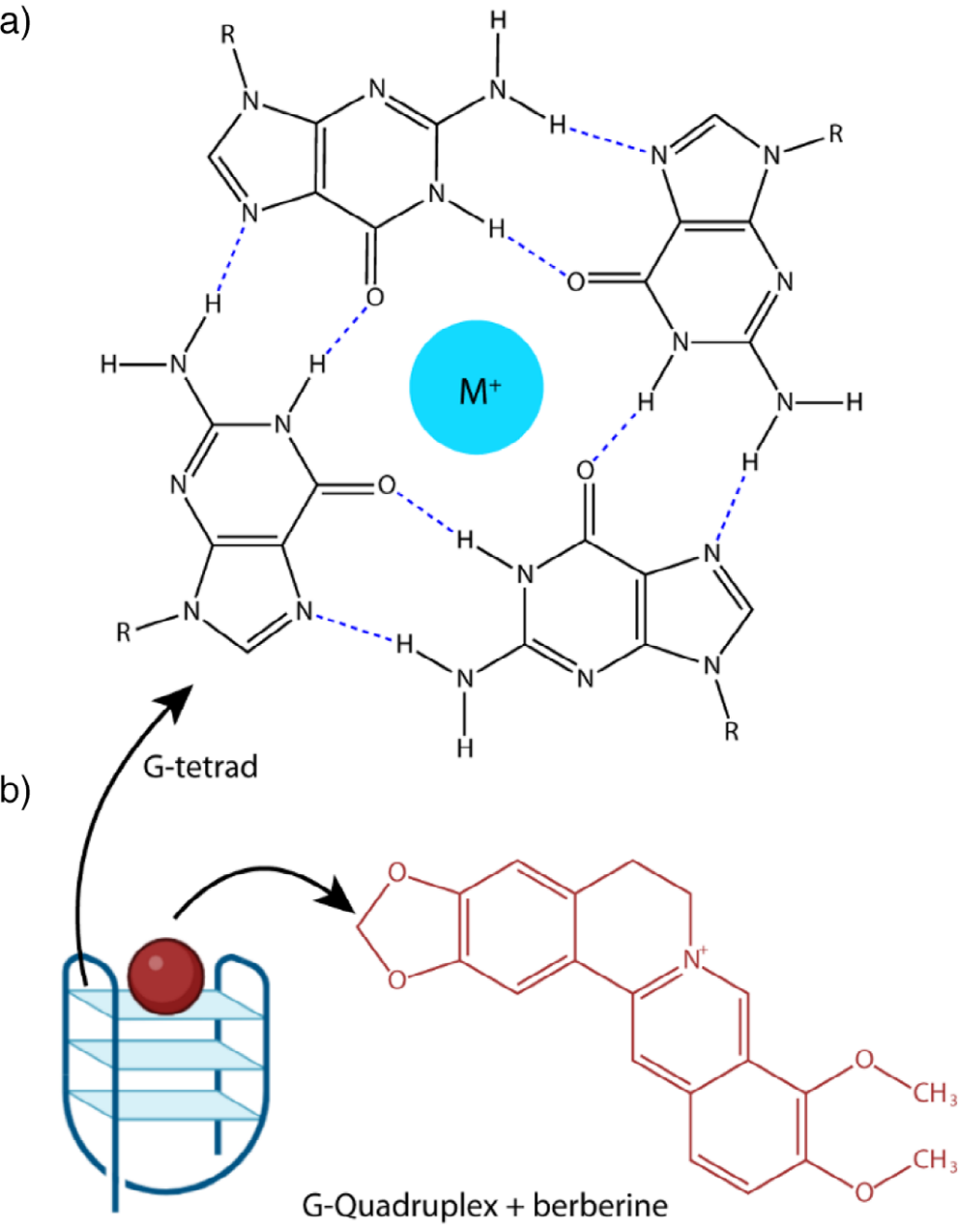
a) Schematic representation of a G‐quadruplex structure depicted by one G‐tetrad of guanine binding via Hoogsteen base pairing. M+ represents a small stabilizing metal cation (K+ or Na+). b) Chemical structure of the natural isoquinoline plant alkaloid berberine. The figure was created using ChemDraw and BioRender.com.

G4s are four‐stranded structures found in guanine‐rich regions, comprising π‐π stacked G‐tetrads with each tetrad consisting of four guanines stabilized by Hoogsteen hydrogen bonds with a centrally coordinated monovalent metallic ion (K^+^ or Na^+^) (Figure 1a).^[^ ^14^, ^15^ ^]^ So far, detailed insight into G4 structure and function in the context of a biologically relevant background has been elusive. In particular, obtaining structural data in vivo has remained challenging for modern structural biology, mainly due to technical limitations.

In recent years, significant technological advancements have been made, particularly within the realm of nuclear magnetic resonance (NMR) spectroscopy, to acquire data in vivo. As a result, solution‐state NMR is now routinely used to study nucleic acids and their interactions with other molecules directly inside living cells,^[^ ^16^, ^17^, ^18^, ^19^, ^20^, ^21^, ^22^, ^23^, ^24^ ^]^ but the approach is limited to small rapidly tumbling assemblies given solution‐state's intrinsic size limitations.^[^ ^25^ ^]^ To overcome these limitations, attention has turned to Magic‐Angle‐Spinning (MAS) solid‐state NMR (ssNMR) which enables studies of protein‐DNA^[^ ^26^ ^]^ and RNA^[^ ^27^, ^28^ ^]^ complexes or probing ion binding to G‐quartets.^[^ ^29^, ^30^ ^]^ Dynamic nuclear polarization (DNP)^[^ ^31^ ^]^ dramatically increases ssNMR sensitivity by transferring radical electron spin polarization to NMR active nuclei, not only facilitating in vitro studies on DNA^[^ ^26^, ^32^ ^]^ but also enabling the elucidation of the structures and functions of large protein assemblies and nucleic acids (NAs) in vivo.^[^ ^33^, ^34^, ^35^, ^36^, ^37^, ^38^ ^]^

The low abundance of mtDNA/RNA inside the cells, which is even magnitudes lower for G4 structures,^[^ ^9^, ^10^ ^]^ their localization within large and complex sub‐organelle assemblies,^[^ ^39^, ^40^ ^]^ and potential spectral overlap with signals arising from nuclear nucleic acids poses a formidable challenge for NMR both in terms of sensitivity and resolution. Here, we employed DNP‐ssNMR to probe the presence of G4 structures by capturing changes in nucleic acid base‐pair interactions upon treatment of intact isolated mitochondria with berberine (Figure 1b). This low‐toxicity natural alkaloid compound is cell‐permeable, highly fluorescent, and exhibits a strong affinity for binding and stabilizing G4 structures,^[^ ^41^, ^42^, ^43^ ^]^ in comparison to duplex DNA.^[^ ^44^, ^45^, ^46^ ^]^ Treatment of mitochondria with berberine triggered spectral changes in nucleic acid sugar and base regions and led to an increase in intensity for correlations arising from Hoogsteen hydrogen bonds observed to interface with berberine upon complexation with G4s in vitro.^[^ ^47^ ^]^ Together, these changes constitute the first evidence of mitochondrial G4 formation in vivo. More broadly, the approach detailed here demonstrates the utility of high‐field DNP‐ssNMR in studying nucleic acid‐ligand interactions in complex biological environments with unprecedented sensitivity and resolution.

## Results

### Isolation of Intact and Functional Mitochondria from Cells

Studying the effect of berberine treatment on mitochondrial nucleic acids (NAs) by DNP‐enhanced ssNMR required the development of a workflow yielding large amounts of intact and functional mitochondria. After optimization, we settled on the following procedure to extract mitochondria: HeLa cells were disrupted by the nitrogen cavitation,^[^ ^48^ ^]^ subjected to multiple rounds of centrifugation, and subsequently separated via a discontinuous Percoll gradient. Western blot data indicated a 4‐fold increase in VDAC (an exclusively mitochondrial protein) abundance with a fluorescent‐based Ca^2+^‐uptake kinetics assay and cryogenic electron microscopy (cryo‐EM) images confirmed that the isolation technique retained both mitochondrial function and structure (Figure S1).

### DNP‐enhancement and Polarization Agent Distribution in Mitochondria

**Figure 2:**
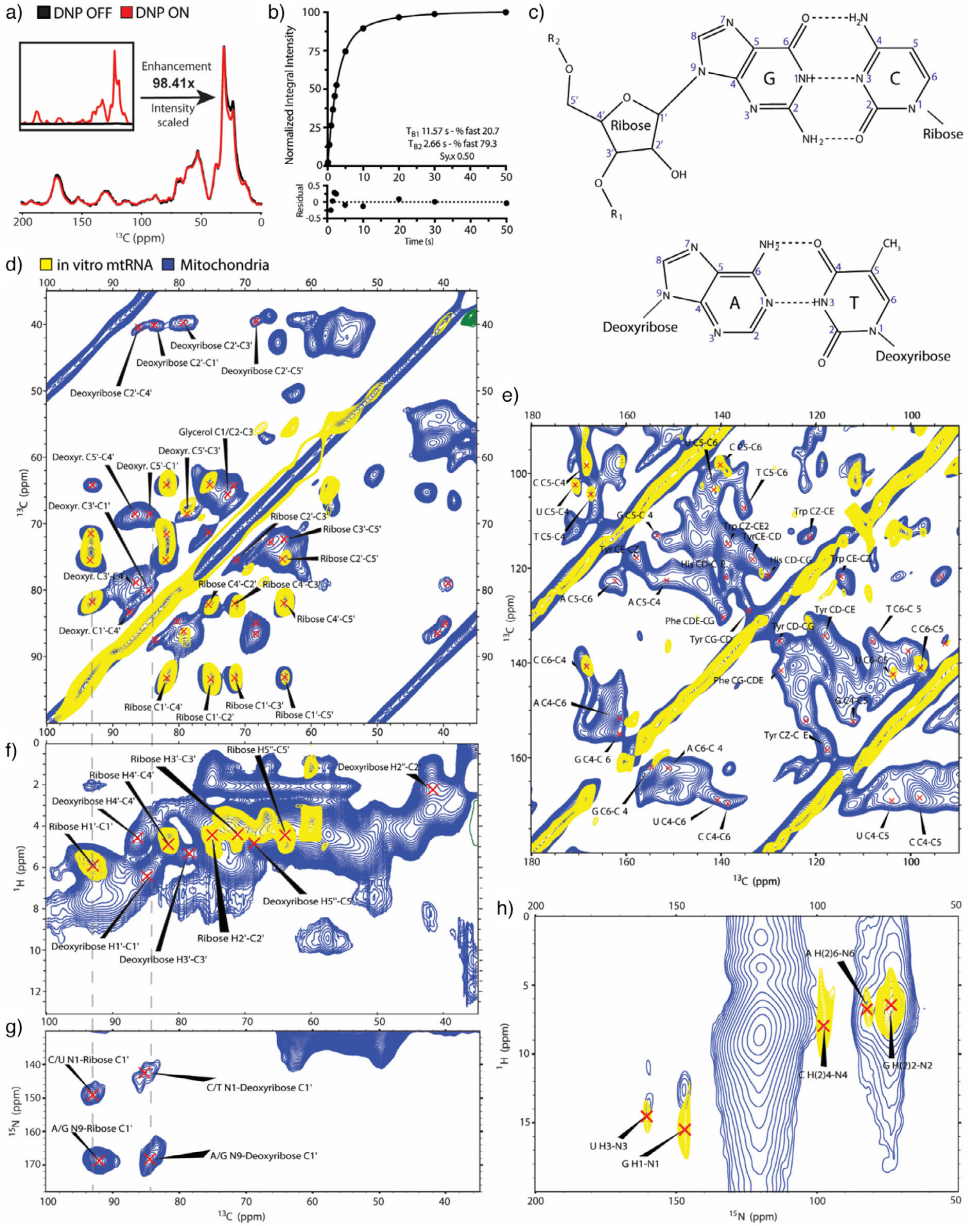
^13^C, ^15^N‐isotopically labeled mitochondria (blue) and isolated mtRNA (yellow) treated with 30 mM SNAPol‐1 were measured under 800 MHz/527 GHz DNP conditions (100 K, 10 kHz MAS). a) Adiabatic ^13^C cross‐polarization (CP) experiments of intact mitochondria with DNP switched ON (red) and OFF (black). The DNP enhancement was determined by comparing signal intensities of individual spectra–DNP ON/OFF. b) Polarization buildup curve (T_B_) of SNAPol‐1 mixed with mitochondria derived from ^1^H‐^13^C CP measurements (see Table S1 for errors). c) Chemical representation of Watson‐Crick base pairing between guanosine‐cytidine (G‐C) and adenosine‐thymidine (A‐T) with the numbering of individual atoms. ^13^C‐^13^C PDSD ssNMR spectra of the sugar d) and base e) region of intact mitochondria (blue) and isolated mtRNA (yellow) with assigned peaks. ^1^H‐^13^C FSLG HETCOR f) and ^13^C‐^15^N DCP g) ssNMR spectra with C1′ sugar correlations shown for ribose and deoxyribose (gray lines). h) ^1^H‐^15^N FSLG HETCOR ssNMR spectra of NA base paring and amino groups correlations. Individual peak assignments can be found in SI Tables S2–S4.

To visualize correlations arising from mitochondrial nucleic acids, an indiscriminate ^13^C,^15^N‐labeling strategy was used.^[^ ^49^, ^50^ ^]^ Isotope‐enriched mitochondria were isolated from adherent HeLa cells cultured in ^13^C, ^15^N‐enriched medium. The mitochondria were then mixed with “DNP juice” (see Supporting Information materials and methods),^[^ ^31^, ^51^ ^]^ rapidly packed into a 3.2 mm sapphire DNP‐magic angle spinning rotor, and plunged frozen in liquid nitrogen.^[^ ^52^, ^53^, ^54^ ^]^ The DNP polarization agent SNAPol‐1^[^ ^51^ ^]^ induced a uniform ^13^C signal enhancement of 98 ± 5 times at 800 MHz/527 GHz (Figure 2a) in line with a ^15^N enhancement of 99 ± 7 for amide backbone signals (Figure S2).

Similar to previous studies, penetration of SNAPol‐1 into mitochondria was assessed by performing ^13^C‐detected cross‐polarization saturation recovery measurements to determine the ^13^C signal buildup time (T_B_).^[^ ^34^, ^54^, ^55^, ^56^, ^57^ ^]^ The T_B_ curve was well explained by a biexponential fit with both a short and a long component (Figure 2b; Table S1). While the short component was longer in mitochondria than what was previously observed in whole cells (2.7 vs 1.4 s), it accounted for a significantly larger share of the fit (79.3 vs 57%). Interestingly, the longer mitochondrial T_B_ value was nearly half of the value observed in whole cells (11.6 s vs 22 s).^[^ ^54^ ^]^ Together, the results indicate that a large proportion of SNAPol‐1 entered mitochondria, albeit with some SNAPol‐1 remaining either embedded in the extra‐mitochondrial matrix or sequestered at the organelle's periphery despite its porous nature. The shorter time constant of the long component can be attributed to mitochondria's small size, which allows for more efficient spin‐diffusion from the periphery into the lumen than would be the case in whole intact cells. Overall, the fractionation and the SNAPol‐1 treatment approach yielded a weighted absolute carbon enhancement sigma of 337 ± 6, thus making the investigation of mitochondrial nucleic acids in situ possible.^[^ ^58^ ^]^

### DNP‐ssNMR of Nucleic Acids Inside Intact Mitochondria

We acquired 2D ^13^C‐^13^C proton‐driven spin‐diffusion (PDSD) spectra^[^ ^59^ ^]^ of fully ^13^C, ^15^N‐labeled extracted mitochondria to observe nucleic acid correlations and possible Watson‐Crick base pairing (Figure 2c) with improved sensitivity and resolution. The spectral region from 35 to 100 ppm, corresponding to nucleic acids sugar correlations, contained well‐resolved signals, with resolution in certain instances superior to what was observed previously by in‐cell DNP‐ssNMR in whole cells (Figure 2d).^[^ ^34^ ^]^ To confirm that the sugar correlations indeed arose from nucleic acids, we recorded DNP‐ssNMR data on fully ^13^C,^15^N‐labeled mtRNA that was extracted from labeled intact mitochondria. The concentration of the mtRNA in this DNP sample was 87 ± 5 µg (Figure S3), and the associated ^13^C signal enhancement (93.5 ± 11, Figure S4) was comparable to our DNP data using intact mitochondria. 2D ^13^C‐^13^C PDSD spectra of mitochondrial (blue) and isolated mtRNA (yellow) were nearly identical in the sugar region (Figure 2d). Due to the remarkable spectral resolution, we could distinguish between deoxyribose (from DNA) and ribose (from RNA) signals in the mitochondrial preparation and fully assign correlations using a combination of literature chemical shift values and transferring assignments from the in vitro mtRNA DNP‐ssNMR spectrum (for the chemical shift values, see SI Table S2).^[^ ^60^, ^61^ ^]^ Additionally, cross‐peaks for carbons (C4‐C5‐C6) originating from individual bases were readily distinguishable in the spectral region between 90–180 ppm (Figure 2e). In the in vitro mtRNA spectrum, cross‐peaks for C5‐C4 (cytosine and uracil) were well resolved. Cumulatively, in both samples, correlations corresponding to pyrimidine bases (cytosine, uracil, thymine – for the chemical shift values, see Table S3) were more pronounced and resolved than those arising from purines (adenine, guanosine), which may reflect the tendency of the former to adopt more rigid conformations upon base pairing.^[^ ^62^ ^]^

Intra‐nucleic acid proton carbon correlations in both samples were probed by applying ^1^H‐^13^C frequency switched Lee‐Goldburg heteronuclear correlation experiments (FSLG HETCOR) (Figure 2f).^[^ ^63^ ^]^ Chemical shifts in the sugar region (35–100 ppm for carbon and 2–6.5 ppm for hydrogen) matched those expected for ribose and deoxyribose moieties. However, the line widths in the proton (^1^H) dimension arising from the intact sample were broad (>2ppm), making individual peak assignments challenging (for chemical shift position, see Table S4). Nevertheless, some peaks could be assigned as originating predominately from cytosine and uracil (Figure S5). Additionally, we performed a ^13^C‐^15^N double cross‐polarization (DCP) experiment^[^ ^64^, ^65^ ^]^ to probe sugar‐base connections. The ^13^C‐^15^N DCP spectrum revealed correlations matching literature assignments for sugar (C1′) and nitrogenous base (N1/N9) connections (Figure 2g; Figure S6 and Table S4). It is highly probable that the inherently lower sensitivity of the DCP experiment precluded visualization of additional nitrogen–carbon base correlations.

As demonstrated before,^[^ ^33^ ^]^ we probed inter‐nucleic acid hydrogen bond interactions by acquiring a series of ^1^H‐^15^ N FSLG HETCOR DNP‐ssNMR measurements, with increasing ^1^H‐^15^N contact times to detect longer distance dipolar spin–spin interactions.^[^ ^63^ ^]^ Subsequently, our spectral analysis focused on changes in imino proton chemical shifts, given their sensitivity to and direct involvement in base‐pairing interactions.^[^ ^66^, ^67^, ^68^ ^]^ The first experiment, which probed for short‐range connections, revealed signals originating from proton‐nitrogen pairs on guanine N1‐H1 (147 ppm; 10–15 ppm) and uracil/thymine N3‐H3 (160 ppm; 10–15 ppm) (Figure 2h, blue). These values revealed the presence of both Watson‐Crick (12–14 ppm) and Hoogsteen‐based (10–12 ppm) structures in intact mitochondria. A subsequent ^1^H‐^15^N FSLG HETCOR experiment probing for long‐distance connections was devoid of additional correlations. However, a detailed analysis was precluded due to the presence of intense amide backbone signals and spinning sidebands because indiscriminate isotope labeling occluded parts of the spectrum. Indeed, an identical set of experiments on extracted mtRNA readily revealed NH_2_ correlations belonging to cytosine, adenine, and guanine (60 to 100 ppm for nitrogen and 5 to 9 ppm for protons) due to the absence of a peptide backbone signal.

### Bound Berberine Affects Nucleic Acid Sugars Inside Mitochondria

Having established a workflow to visualize nucleic acids with unprecedented spectral sensitivity and resolution within intact mitochondria, the focus shifted to detecting the presence of G4s. We reasoned that doping mitochondria with berberine would increase the intensity of Hoogsteen base pair imino signals due to G4 structure stabilization.

**Figure 3:**
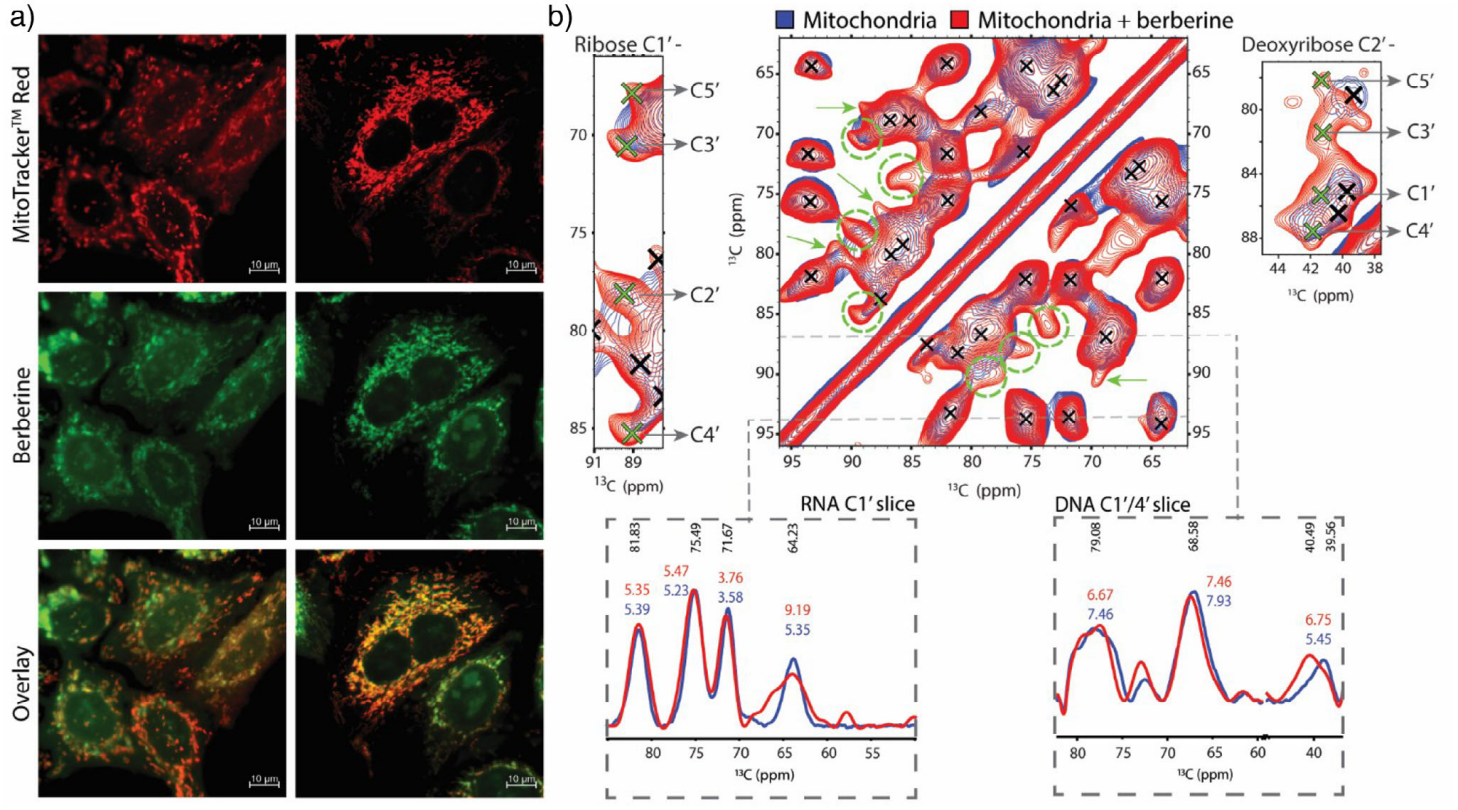
a) Confocal microscopy of live HeLa cells treated with 10 µM berberine (green) for 14 hours, stained before measurement with MitoTracker^TM^ Red (red) for mitochondria visualization. b) ^13^C‐^13^C PDSD DNP‐ssNMR spectra of sugar region of ^13^C, ^15^N‐isotopically labeled mitochondria (blue) and treated with berberine (red) were mixed with 30 mM SNAPol‐1 and measured at 800 MHz/527 GHz DNP conditions (100 K, 10 kHz MAS). Left and Right depict zoomed regions for ribose C1′ and deoxyribose C2′ correlations, respectively. New cross‐peaks arising from NA interactions with berberine are marked in green. Bottom: 1D slices (gray lines) of the ^13^C‐^13^C PDSD DNP‐ssNMR spectrum for mtDNA and mtRNA with (red) and without berberine (blue) for assigned peaks and their line width.

The maximal concentration of berberine that could be used without inducing cell death and proliferation was determined by titrating HeLa cells with berberine and subsequently observing changes in cellular viability via acquiring microscopy images (Figure S7). Utilizing confocal microscopy allowed us to also leverage berberine's autofluorescence properties for simultaneous detection of its penetration into the mitochondrial lumen (Figure 3a).^[^ ^69^ ^]^

Fully labeled ^13^C‐^15^N mitochondria were incubated with 30 µm (non‐isotope enriched) berberine for 30 min before mixing with DNP juice and plunge freezing. Subsequently, we repeated the 2D experiments described above on the berberine‐treated mitochondria sample. The ^13^C‐^13^C PDSD experiment of berberine‐treated ^13^C, ^15^N‐labeled mitochondria (red) was overlaid with that of the untreated sample (blue) (Figure 3b, Figure S8). The berberine‐treated spectrum, particularly the nucleic acids’ sugar region (35–100 ppm), contained numerous new correlations (highlighted by green circles and crosses) and some previous correlations undergoing spectral broadening (indicated by green arrows). While the emerging peaks largely overlapped with those previously observed, analysis of less crowded regions revealed multiple maxima for ribose C1′ and deoxyribose C2′ (Figure 3b). This multiplicity strongly suggested that the berberine treatment either increased the abundance of previously minor conformers or rigidified conformers previously invisible because they sampled large conformational spaces. We note that a similar comparison was precluded for the ^1^H‐^13^C FSLG HETCOR due to peak broadening and spectral crowding (Figure S9). The ^13^C‐^15^N DCP experiment (Figure S10) was populated with the same carbon‐ nitrogen base‐sugar connections as the untreated mitochondria sample.

To better understand the spectral changes that were evident in the 2D ^13^C‐^13^C PDSD spectra induced by berberine treatment, we extracted 1D slices (gray lines, Figure 3b). These showed that for many resonances, the NMR line width in both samples (treated/untreated) remained largely the same. Notably, in both samples, mtDNA correlations exhibited broader line widths (5.5–7.5 ppm) than mtRNA signals (3.5–5.5 ppm), except for the C1′‐C5′ correlation. The broader line widths evident for mtDNA suggest that it samples multiple conformations within nucleoids.^[^ ^70^ ^]^ The observed decrease in line width for mtRNA may also result from more conformationally homogeneous sugar structures, which is in line with literature suggesting that mtRNA is compactly stored in granules.^[^ ^39^ ^]^ In the treated sample, the line widths of both mtDNA and mtRNA C1′‐C5′ correlations increased from 5.5 to 6.8 ppm and from 5.4 to 9.2 ppm, respectively (Figure 3b). In addition, a change in chemical shift for mtDNA C2′‐C5′ correlation of ≈1 ppm (from 39.5 to 40.5 ppm, Figure 3b) was evident. These spectral changes were expected, given in vitro structural data indicating that sugar moieties experienced chemical‐shift modulations upon berberine binding to the G‐quadruplex structure due to steric hindrance.^[^ ^47^, ^71^ ^]^

### Impact of Berberine on DNA/RNA Base Pairing Within Mitochondria

**Figure 4:**
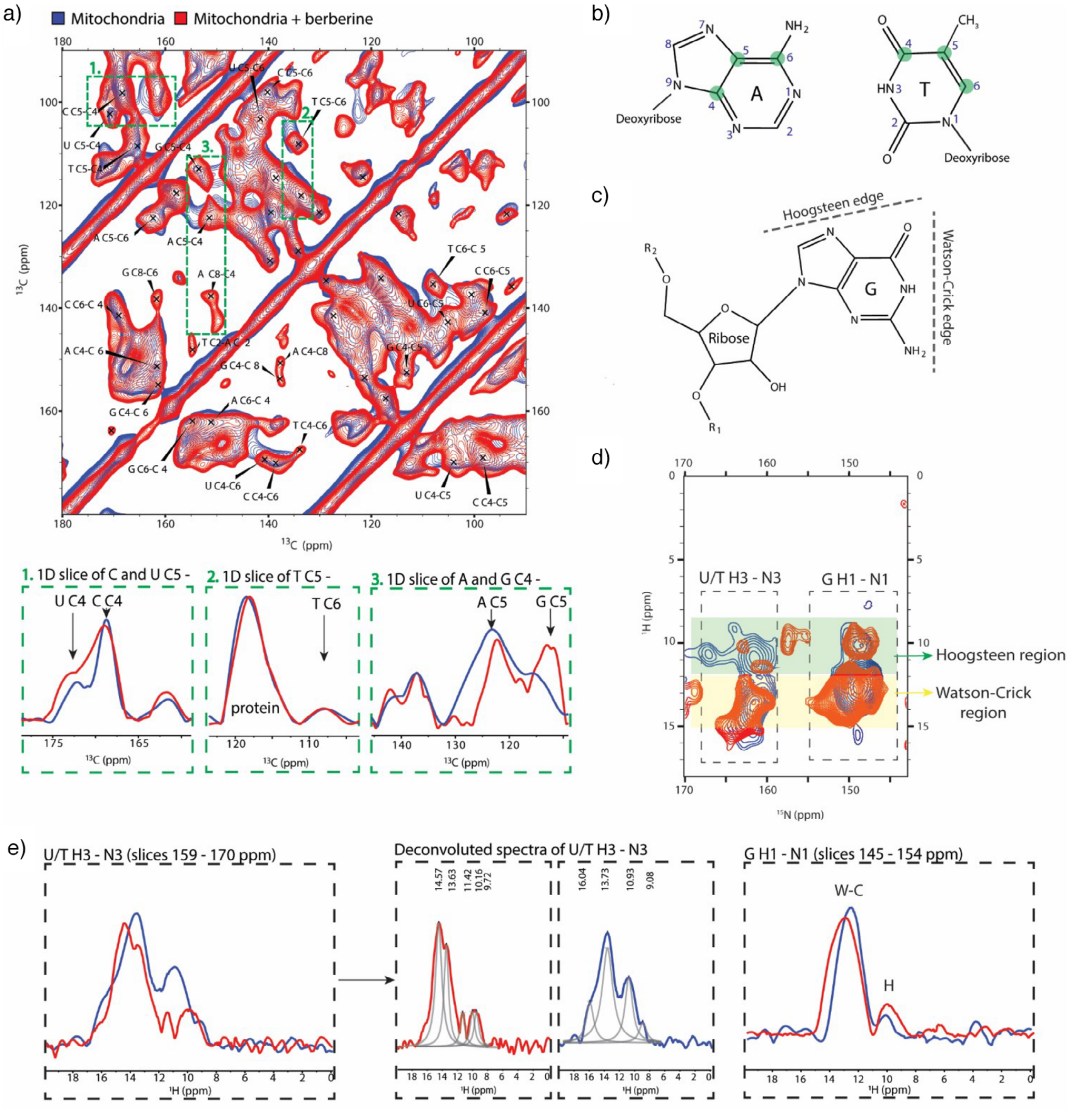
^13^C, ^15^N‐isotopically labeled mitochondria with (red) and without berberine (blue) were treated with 30 mM SNAPol‐1 and measured at 800 MHz/527 GHz DNP conditions (100 K, 10 kHz MAS). a) ^13^C‐^13^C PDSD spectra of the NA base region with peak assignments and 1D projections for individual carbon atoms in nucleotide bases (green squares). b) Chemical representation of purine (A) and pyrimidine (T) bases with numbering and highlighted carbon atoms (green) observable in the ^13^C‐^13^C PDSD spectrum. c) Chemical representation of guanosine residue with marked edges for Watson‐Crick and Hoogsteen interactions. d) ^1^H‐^15^N FSLG HETCOR DNP‐ssNMR spectra with highlighted U/T H3‐N3 and G H1‐N1 Watson–Crick and Hoogsteen base pair regions. e) 1D projection of ^1^H‐^15^N FSLG HETCOR spectra for U/T H3‐N3 (slices 159–170 ppm) and their deconvolution to individual peaks and G H1‐N1 (slices 145–154 ppm) with integrated regions for Watson–Crick (W–C) and Hoogsteen (H) base pairing, revealing that the Hoogsteen base‐pair signal intensity increases while the Watson‐Crick signal intensity decreases.

Having obtained evidence that berberine binding induced spectral changes in the sugar region, we subsequently assayed changes in the nucleic acid base region (90–180 ppm) of the 2D ^13^C‐^13^C PDSD spectra (Figure 4a).^[^ ^72^, ^73^ ^]^ Visible correlations for nucleic acids in the spectra are highlighted with green circles (Figure 4b). Spectral changes were observed in the treated sample (red), including changes in resolution, chemical‐shift perturbations, and the emergence of new correlations. Importantly, protein signals remained unchanged in both sample types (Figure 4a).

To gain a clearer understanding of the observed spectral changes, we extracted 1D projections from the ^13^C‐^13^C PDSD spectrum (Figure 4a). The first 1D projection (95–105 ppm) captured the pyrimidine C5‐C4 (uracil and cytosine) connection. The projections were quite similar, albeit with slight changes in peak width. The same was evident for cross‐peaks arising from thymine C5‐C6. While adenine and guanine C4 and C6 correlations were indistinguishable due to peak broadening, 1D projections allowed to discriminate between adenine and guanine C4‐C5 connections (148–156 ppm).^[^ ^74^, ^75^ ^]^ These correlations were more resolved in the berberine‐treated sample and experienced chemical shift perturbations. Additionally, the guanine C4‐C5 peak intensity increased significantly, which is indicative of an increase in the number of guanines adopting similar structurally homogenous and rigid conformation. Subsequently, we conducted a ^1^H‐^15^ N FSLG HETCOR experiment to investigate potential changes in nucleic acid base pairing, again focusing on Watson–Crick and Hoogsteen interactions (Figure 4c). The spectral pattern of ^1^H‐^15^N FSLG HETCOR spectra looked similar (Figure 4d), with strong signals stemming from protein NH backbone and arginine NH_2_ groups (Figure S11). Overlaying treated (red) and untreated (blue) spectra revealed that the signal of several correlations, namely guanine H1‐N1 (150 ppm in nitrogen) and uracil/thymine H3‐N3 (160 ppm in nitrogen), experienced chemical shift perturbations. 1D projections of imino signals allowed us to estimate changes in individual base paring mode populations (Figure 4e). Deconvolution of uracil/thymine H3‐N3 ssNMR signals revealed the presence of at least four imino peaks, two sampling chemical shift values expected for Watson–Crick (12–14 ppm) and two for Hoogsteen (10–12 ppm) base pairing.^[^ ^68^, ^76^ ^]^ The ratio between the two base pairing modes shifted toward Watson–Crick upon berberine binding. Conversely, the H1–N1 imino signal of guanine showed that berberine binding promoted more Hoogsteen base pairing (1:6.7) compared to Watson–Crick, which was more prevalent in the untreated sample (1:9). Taken together, these observations suggested that the berberine treatment increased the number of G4s (or Hoogsteen based structures) or rigidified their structure thereby rendering them more spectrally visible.

## Discussion

While G‐quadruplexes are established to be essential regulatory elements of the nuclear genome, their distribution and roles in mtDNA/RNA are not well understood.^[^ ^77^ ^]^ Emerging evidence suggests that alternative DNA/RNA structures, like G‐quadruplexes, also play crucial regulatory roles in mitochondria.^[^ ^10^ ^]^ However, direct insight into the presence and role of such structures in vivo has remained elusive.^[^ ^10^ ^]^ Here, we have presented a method that allows studying nucleic acids and their interactions with ligands at atomic resolution by DNP‐ssNMR within intact mitochondria at endogenous concentrations. To enable such studies, we compacted the mitochondrial content of ≈1.4 × 10^8^ HeLa cells into a single 3.2 mm MAS rotor, which maximally accommodates only 1/9 of the number of cells. Together with 100‐fold signal enhancement at 800 MHz by using SNAPol‐1 as a polarization agent, our current approach leads to a signal enhancement of almost three orders of magnitude compared to using conventional ssNMR methods.^[^ ^52^, ^54^ ^]^

To probe the presence of G4 structures in mitochondria, we utilized berberine, a well‐known ligand with low toxicity, good permeability, and high specificity for G4 structures.^[^ ^43^ ^]^ The observed spectral changes (including the appearance of new peaks) for nucleic acid sugar (ribose and deoxyribose) and base (guanine C4‐C5) correlations in the 2D^13^C‐^13^C PDSD data sets strongly indicate that berberine binds to both mtDNA and mtRNA thereby stabilizing previously invisible or lowly populated sugar conformers. Our data would also be consistent with the presence of multiple sugar conformations for deoxyribose and ribose in vivo. Additionally, a closer examination and integration of the 1D projections corresponding to the guanine H1‐N1 imino region of the ^1^H‐^15^N FSLG HETCOR spectrum revealed a sizeable change in the ratio between Watson–Crick and Hoogsteen base pair containing structures for treated mitochondria, hence emphasizing the increased presence of G‐quadruplex structures in mitochondria in vivo after ligand binding.

We have demonstrated that ligand binding events can be directly followed in situ by DNP‐ssNMR and that in vivo mitochondrial spectra exhibit commonalities with previously published in vitro work.^[^ ^41^, ^47^, ^62^ ^]^ Extracting more quantitative data from these spectra may be possible using tailored labeling strategies that enable smaller spectral acquisition windows, resulting in spectra with more sensitivity and resolution. Moreover, further advances in polarizing agent design, DNP probe technology, and subsequent acquisition of DNP‐ssNMR at even higher magnetic fields will yield more sensitive and better‐resolved spectra.

## Conclusion

Taken together, our findings provide valuable insights into the molecular interactions and potential structural stabilizations induced by berberine in mitochondrial nucleic acids. They also pave the way for using DNP‐ssNMR spectroscopy to study these and other nucleic acid interactions in situ, which could enhance our understanding of mitochondrial translation, transcription, and related diseases, ultimately aiding in the development of novel treatments.

## Supporting Information

The authors have cited additional references within the Supporting Information.^[^ ^78^, ^79^, ^80^, ^81^, ^82^ ^]^

## Conflict of Interests

The authors declare no conflict of interest.

## Supporting information



Supporting Information

## Data Availability

The data that support the findings of this study are available from the corresponding author upon reasonable request.
