# Supplementary material for: A DNP‐Supported Solid‐State NMR Approach to Study Nucleic Acids In Situ Reveals Berberine‐Stabilized Hoogsteen Structures in Mitochondria
Source: Angew Chem Int Ed Engl. 2025 Mar 18;64(21):e202424131. doi: 10.1002/anie.202424131 (PMC12087828; doi:10.1002/anie.202424131)
Supplement: Supplementary file 1 — Supporting Information [file ANIE-64-e202424131-s001.docx]

**Supporting Information**

Table of Contents

[Figure S1. S2](#_Toc183550300)

[Figure S2. S3](#_Toc183550301)

[Figure S3. S3](#_Toc183550302)

[Figure S4. S4](#_Toc183550303)

[Figure S5. S4](#_Toc183550304)

[Figure S6. S5](#_Toc183550305)

[Figure S7. S5](#_Toc183550306)

[Figure S8. S6](#_Toc183550307)

[Figure S9. S7](#_Toc183550308)

[Figure S10. S8](#_Toc183550309)

[Figure S11. S8](#_Toc183550310)

[Table S1. S9](#_Toc183550311)

[Table S2. S10](#_Toc183550312)

[Table S3. S11](#_Toc183550313)

[Table S4. S12](#_Toc183550314)

[Materials and Methods S13](#_Toc183550315)


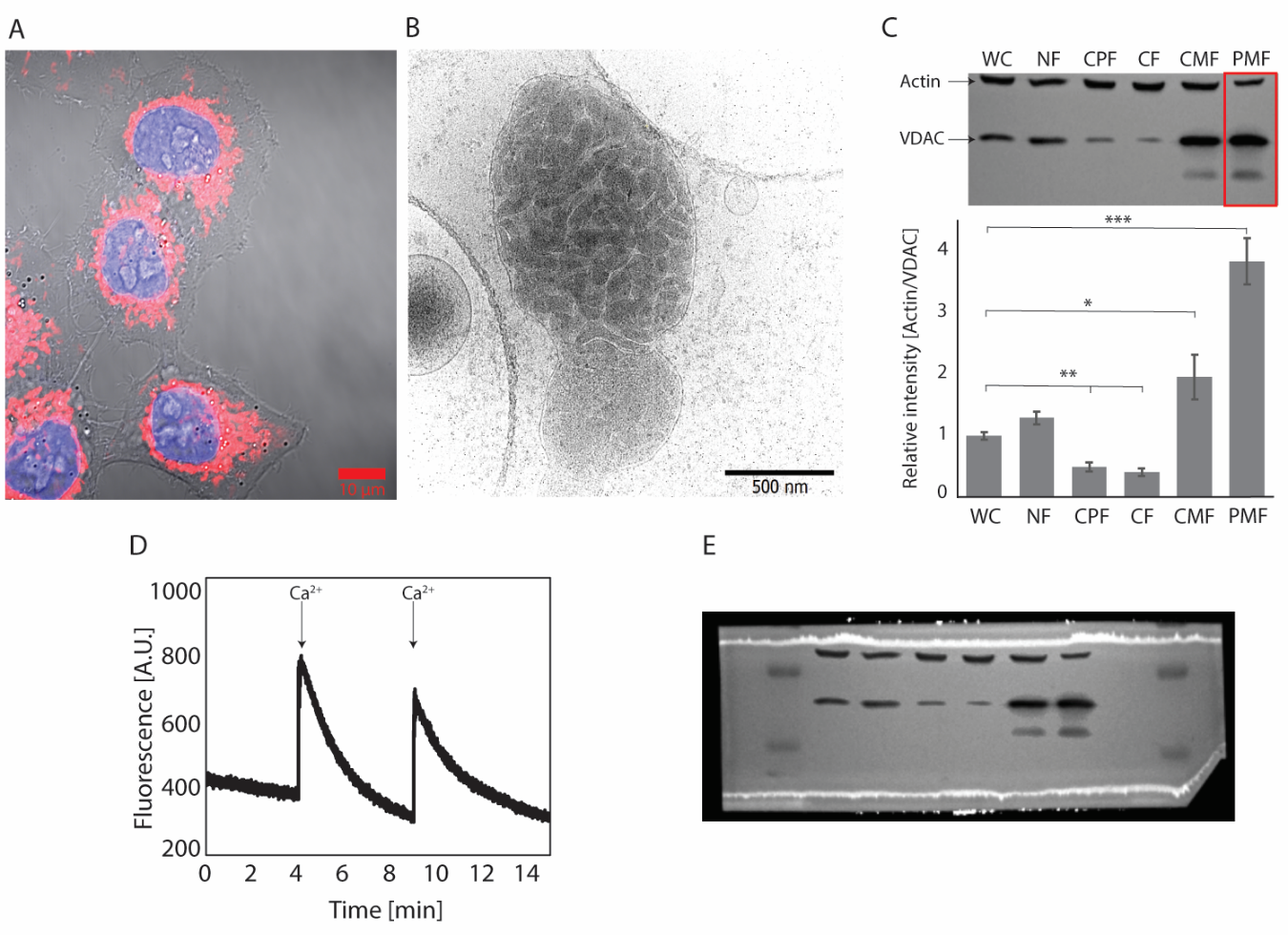


Figure S1.

**(A)** Confocal microscopy of live HeLa cells stained with Hoechst 33342 (blue) to visualize the cell nuclei and MitoTracker^TM^ Red (red) for mitochondria. **(B)** Cryo-electron microscopy image of an intact mitochondrial membrane after isolation from HeLa cell. **(C)** Western blot analysis of individual fractions of HeLa cell lysates (whole cells (WC), nuclear fraction (NF), cytosolic fraction (CF), cytoplasmatic fraction (CPF), crude mitochondria fraction (CMF), and pure mitochondria fraction (PMF)) in each step of the isolation process of mitochondria using antibodies for Actin (control) and VDAC (mitochondrial) protein. Data analysis of Western blots was conducted using both biological and technical triplicates (n=9), with mean values presented alongside standard deviations (SD), T-test, and p-values (*<0.05, **<0.01, ***<0.005). **(D)** Calcium uptake kinetics in isolated pure mitochondria measured using the Calcium
Green-5N fluorescent marker, followed by injection of 10 µM Ca^2+^. **(E)** Full-size Western blot for Figure S1C with chemiluminescence and light channels enabled.


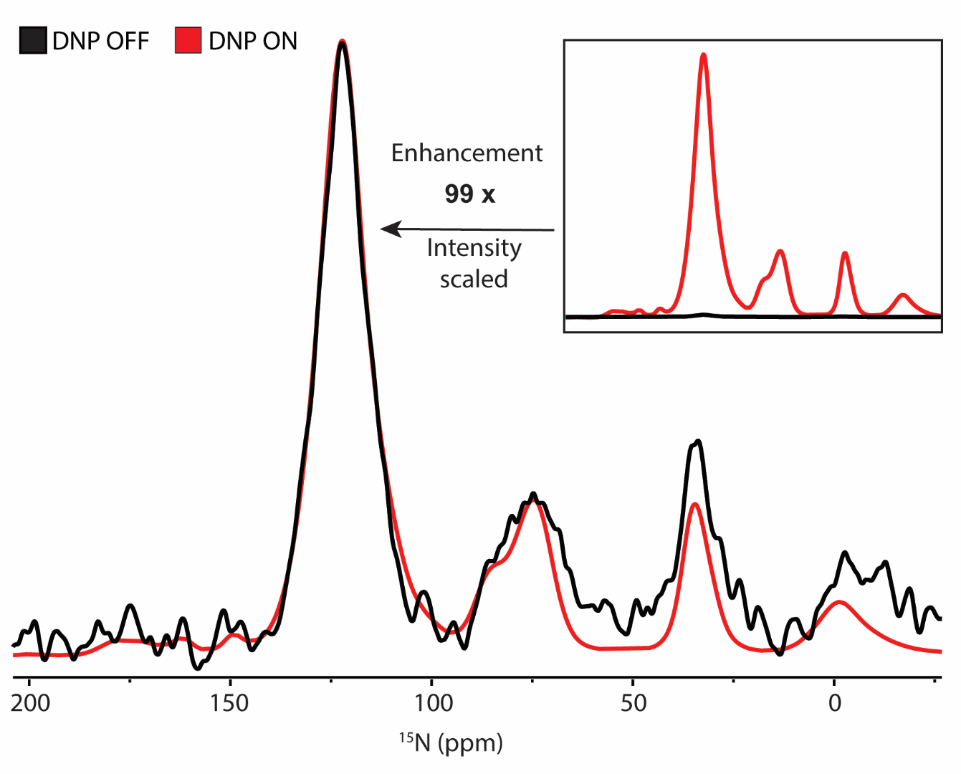


Figure S2.

1D ^1^H-^15^N CP ssNMR spectra of intact ^13^C, ^15^N-isotopically labeled mitochondria treated with 30 mM SNAPol-1. Data were obtained under DNP conditions (100 K, 10 kHz MAS) measured at 800MHz/527GHz. Spectra were recorded with DNP switched ON (red) and OFF (black). Intensity scaling revealed a DNP enhancement factor of 99.


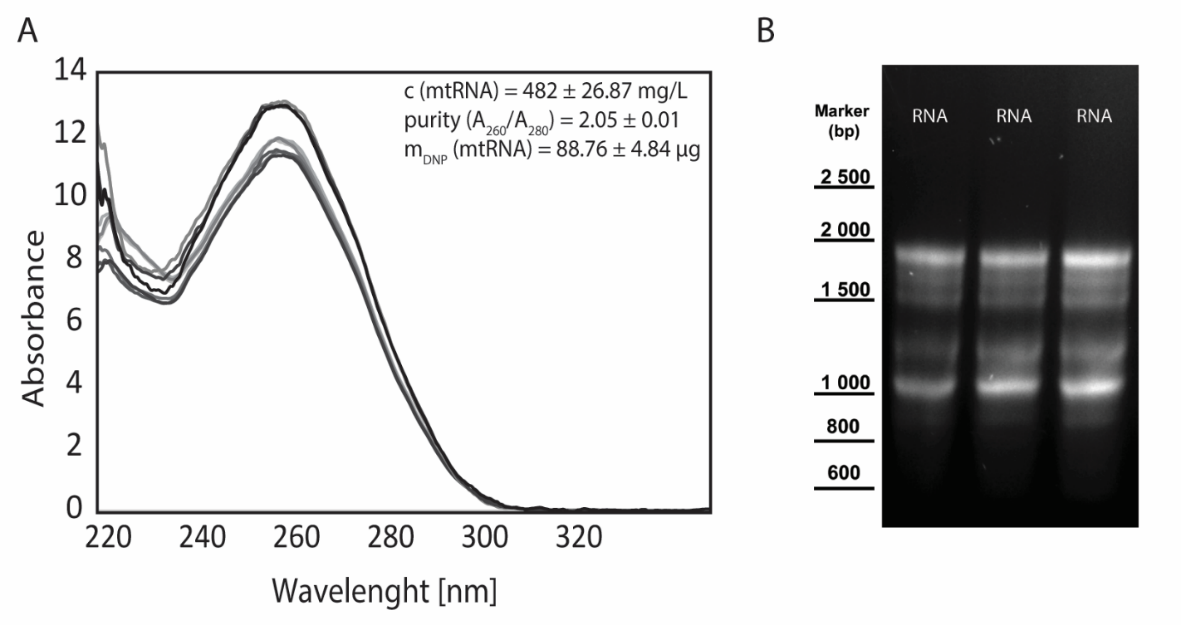


Figure S3.

**(A)** Purity and quantity of isolated mtRNA measured by UV-Vis absorbance spectra in biological and technical replicates (n=9). The concentration of mtRNA within a DNP sample was 87 ± 4.8 μg, and its purity was determined to be 2.01 ± 0.01 by comparison of absorbances A260/A280 (n=9, ±SD provided by NanoDrop spectrophotometer). **(B)** The length of individual mtRNA fragments was determined by 1.2% agarose gel with fragments length from 900 to 2 000 bp.


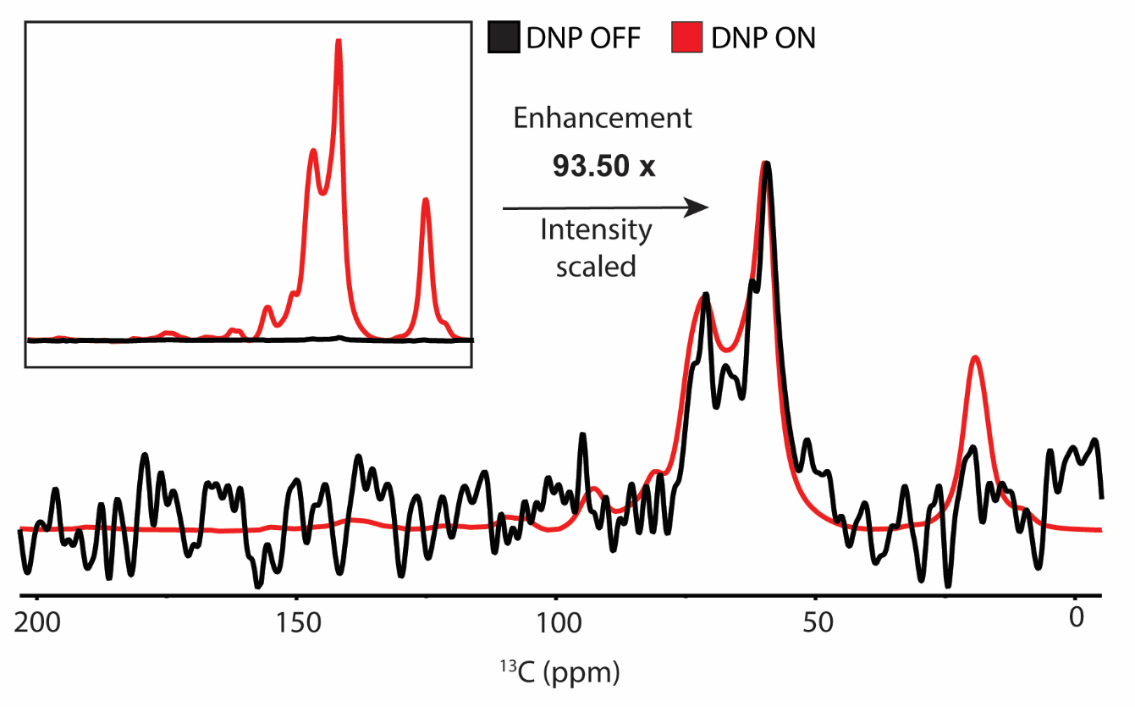


Figure S4.

1D ^1^H-^13^C CP ssNMR spectra of ^13^C, ^15^N-isotopically labeled mtRNA isolated from mitochondrial DNP sample after measurement treated with 30 mM SNAPol-1. Data were obtained under DNP conditions (100 K, 10 kHz MAS, 800MHz/527GHz). Spectra were recorded with DNP switched ON (red) and OFF (black). Intensity scaling revealed a DNP enhancement factor of 93.5.


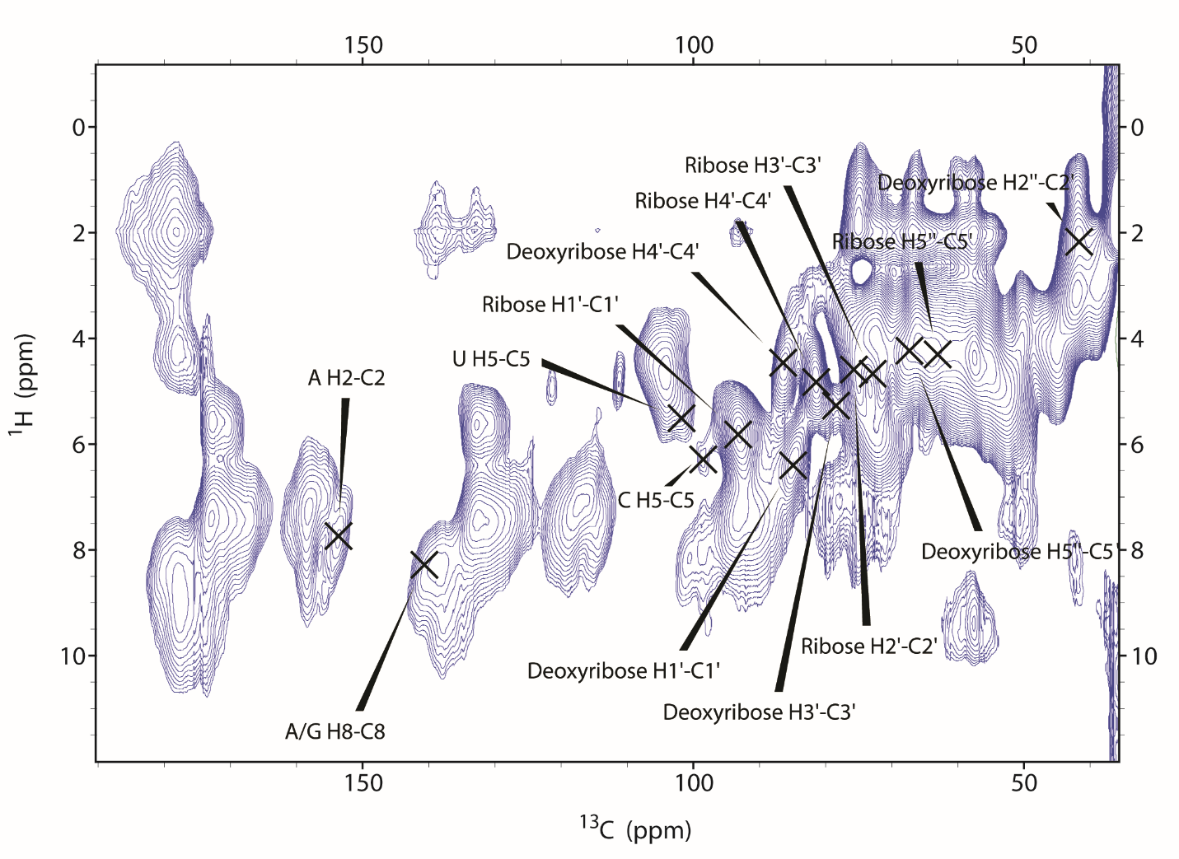


Figure S5.

^1^H-^13^C FSLG HETCOR ssNMR spectrum of intact ^13^C, ^15^N-isotopically labeled mitochondria treated with 30 mM SNAPol-1. Data were obtained under DNP conditions (100 K, 10 kHz MAS, 800MHz/527GHz).


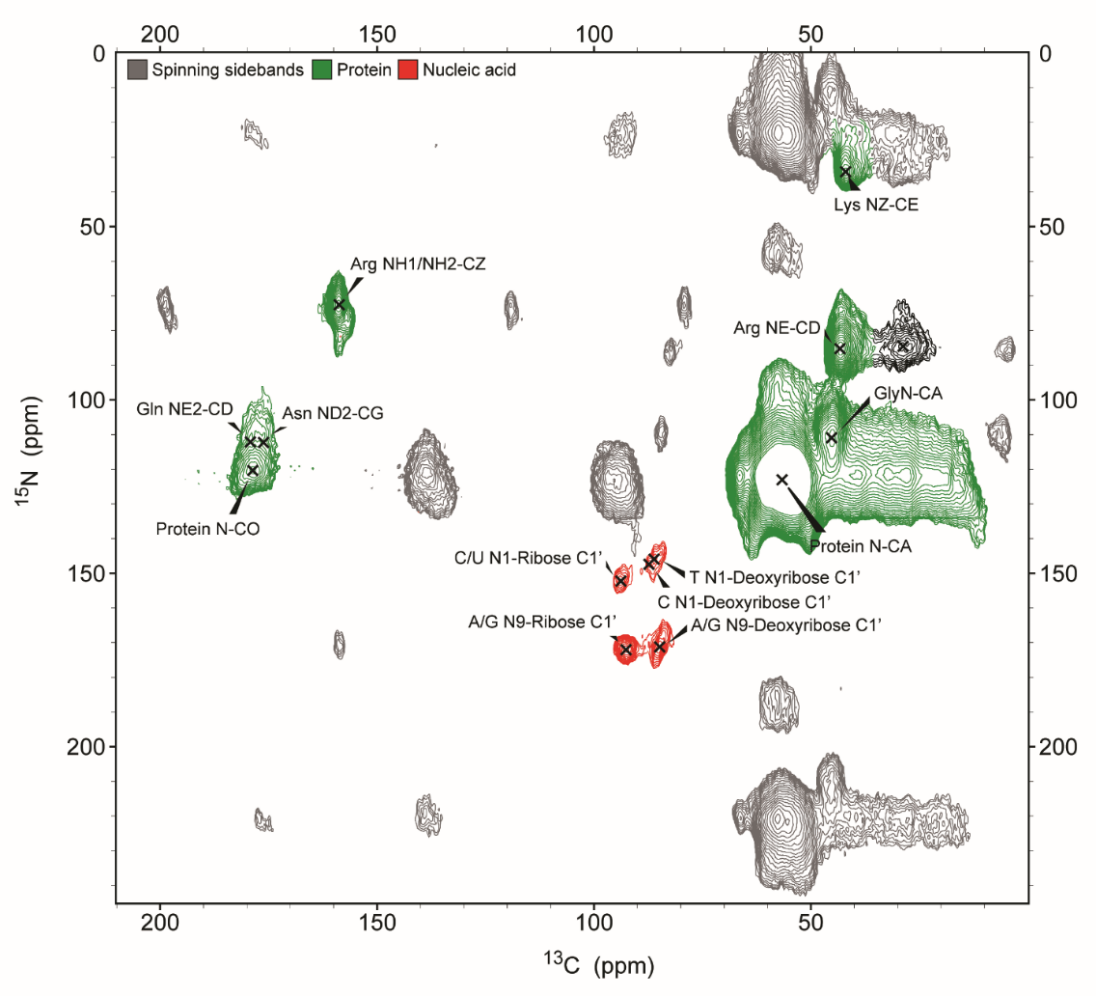


Figure S6.

^13^C-^15^N DCP ssNMR spectrum of intact ^13^C, ^15^N-isotopically labeled mitochondria treated with 30 mM SNAPol-1 that was obtained under DNP conditions (100 K, 10 kHz MAS, 800MHz/527GHz). Color mapping was applied to distinguish individual peaks: spinning sidebands (SSB) were marked in gray, protein signals in green, and nucleic acid signals in red.


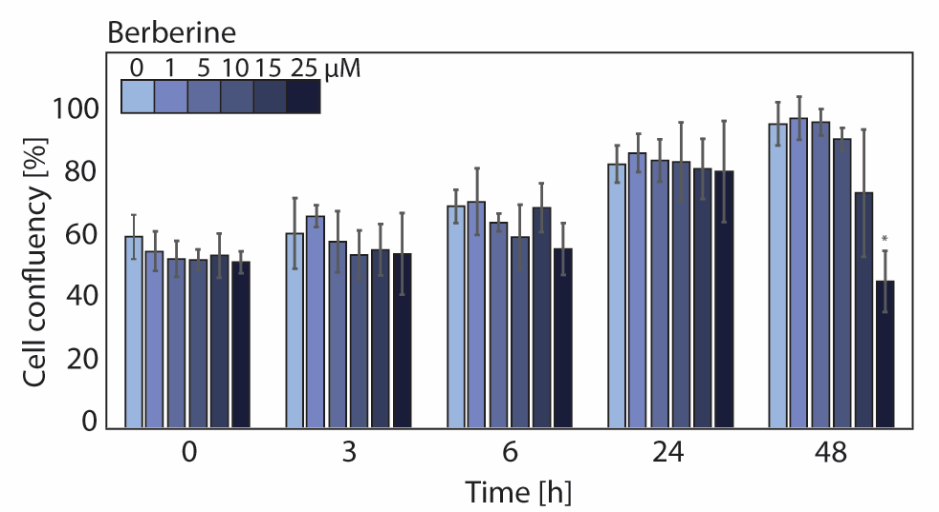


Figure S7.

HeLa cells were treated with berberine at concentrations of 0, 1, 5, 10, 15, and 25 µM for 48 hours. Cell confluency was assessed using microscope images and data were processed by ImageJ based on surface area of cells. The experiment was conducted in biological and technical triplicates (n=9) with mean values presented alongside standard deviations (SD), T-test, and p-values (*<0.05, **<0.01, ***<0.005).


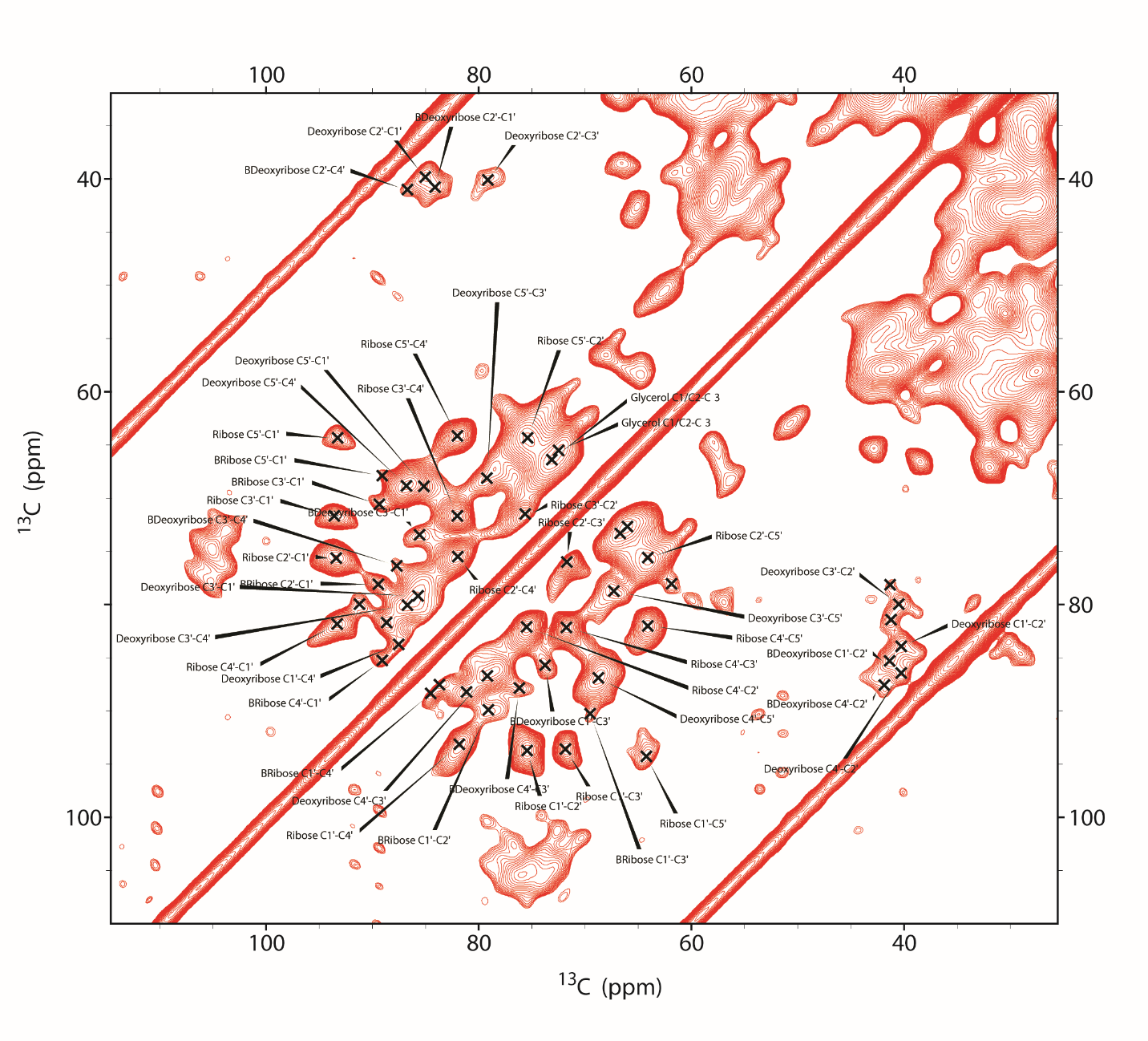


Figure S8.

^13^C-^13^C PDSD DNP-ssNMR spectrum of the sugar region of ^13^C, ^15^N-isotopically labeled mitochondria treated with berberine using 30 mM SNAPol-1 and measured at 800MHz/527GHz DNP conditions (100 K, 10 kHz MAS).


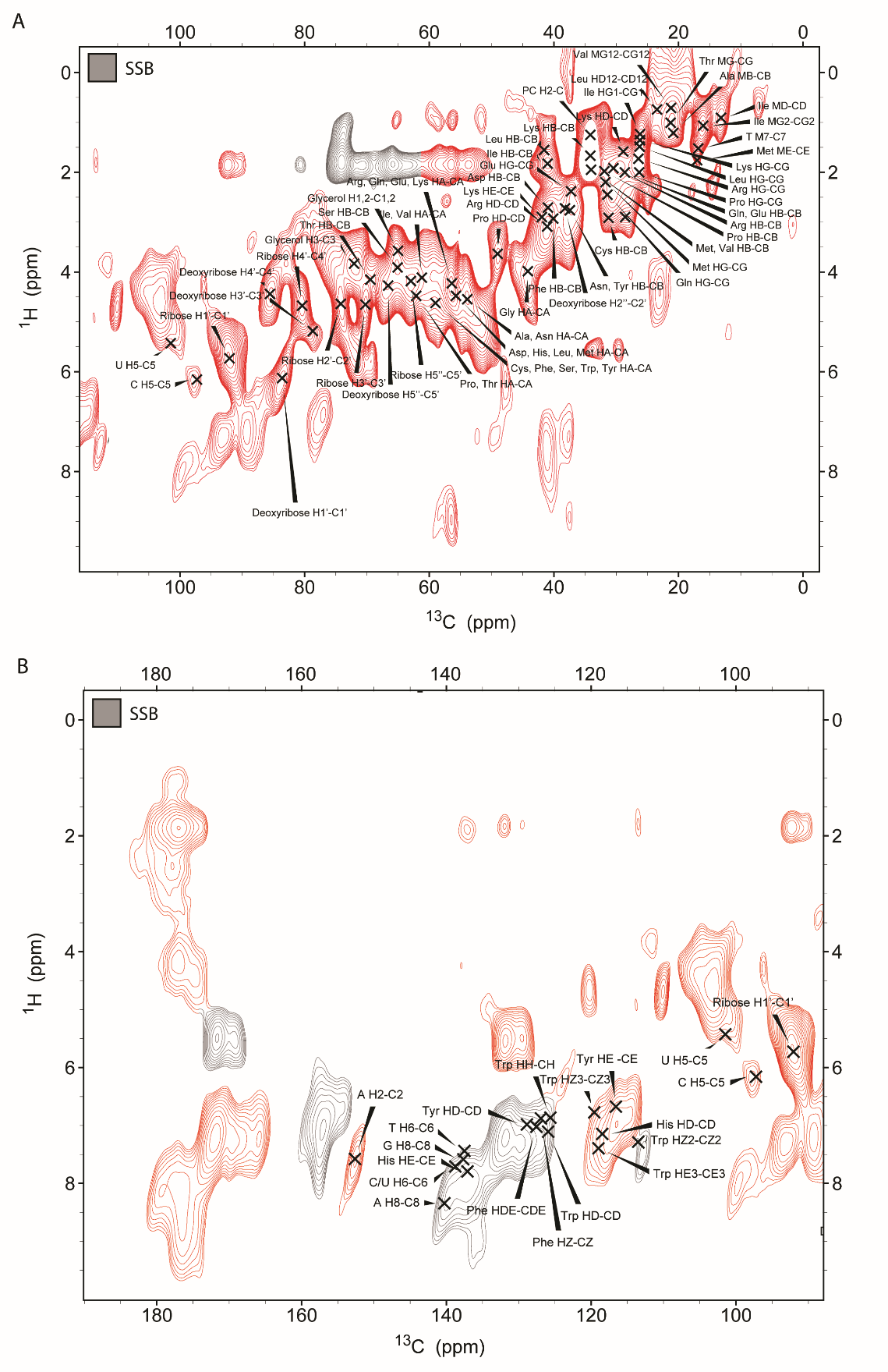


Figure S9.

^1^H-^13^C FSLG HETCOR ssNMR spectrum of sugar **(A)** and base region **(B)** of intact ^13^C, ^15^N-isotopically labeled mitochondria after addition of berberine. The DNP spectrum (100 K, 10 kHz MAS, 800MHz/527GHz) was obtained using 30 mM SNAPol-1.


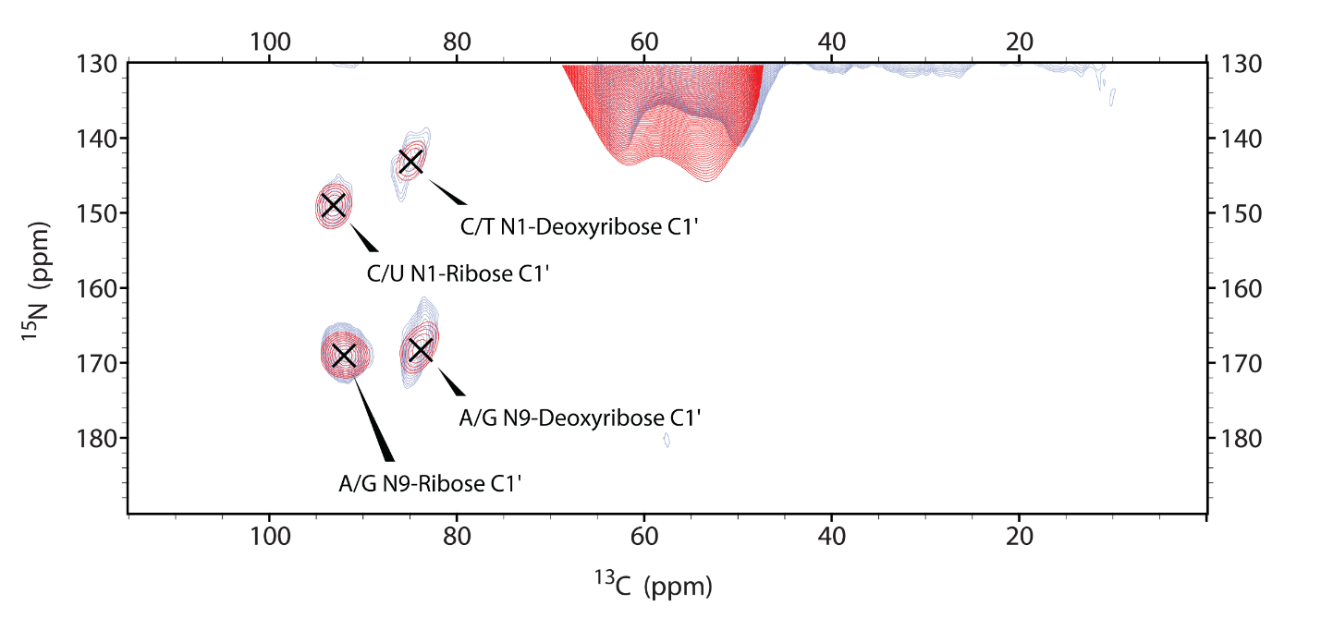


Figure S10.

^13^C-^15^N DCP ssNMR spectra of intact ^13^C, ^15^N-isotopically labeled mitochondria (blue) and mitochondria with berberine (red) were mixed with 30 mM SNAPol-1 and measured under DNP conditions (100 K, 10 kHz MAS) at 800MHz/527GHz.


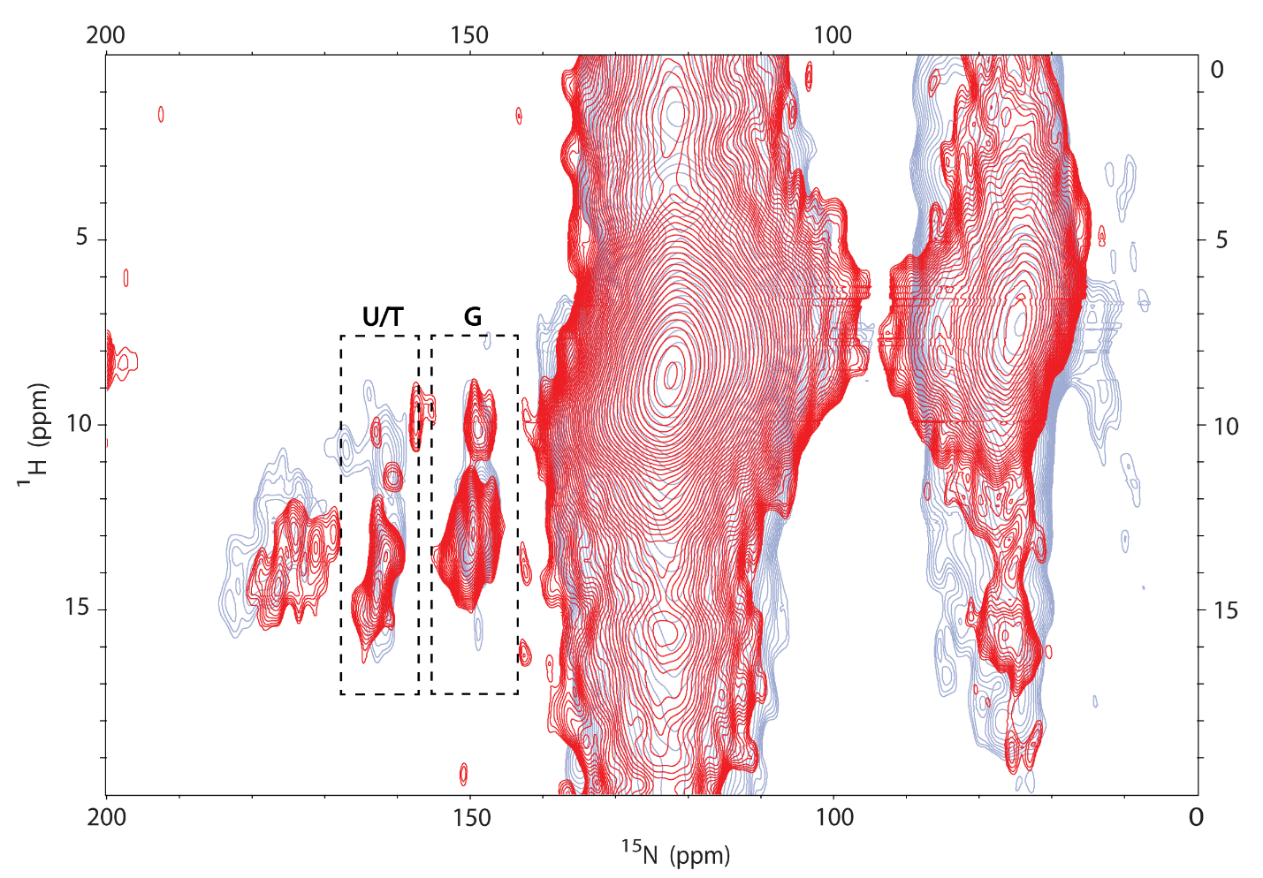


Figure S11.

^1^H-^15^N FSLG HETCOR DNP-ssNMR spectra of intact ^13^C, ^15^N-isotopically labeled mitochondria (blue) and mitochondria with berberine (red), highlighting U/T (uracil/thymine) H3-N3 and G (guanine) H1-N3 base pair regions. DNP samples were mixed with 30 mM SNAPol-1 and measured under DNP conditions (100 K, 10 kHz MAS) at 800MHz/527GHz.

| Time (s) | Normalized Integral Intensity | Error |
| --- | --- | --- |
| 0.005 | 1.03 | 0.015 |
| 0.1 | 2.457 | 0.041 |
| 0.5 | 13.86 | 0.33 |
| 1 | 26.29 | 0.36 |
| 1 | 36.73 | 0.42 |
| 2 | 45.51 | 0.43 |
| 2 | 52.64 | 0.44 |
| 5 | 74.51 | 0.49 |
| 10 | 89.54 | 0.52 |
| 20 | 96.66 | 0.50 |
| 30 | 98.76 | 0.41 |
| 50 | 100 | 0.27 |

Table S1.

^1^H **-**^13^C saturation recovery cross polarization T_B_ data of ^13^C, ^15^N-isotopically labeled mitochondria with associated error. Error was calculated based on publication [54].

| ASSIGNMENT OF ^13^C-^13^C PDSD SPECTRA - SUGARS | | | | |
| --- | --- | --- | --- | --- |
| Correlation | **ω1 (ppm)** | **ω2 (ppm)** | **δ ω1 (ppm)** | **δ ω2 (ppm)** |
| Deoxyribose C2'-C5' | 39.6 | 68.3 | -0.43 | -0.15 |
| Deoxyribose C2'-C3' | 39.8 | 79.6 | -0.20 | -0.04 |
| Deoxyribose C2'-C1' | 40.1 | 83.4 | +0.16 | -0.85 |
| Deoxyribose C2'-C4' | 40.5 | 86.1 | +0.51 | -0.64 |
| Ribose C5'-C4' | 64.2 | 81.8 | +0.05 | -0.13 |
| Ribose C5'-C1' | 64.2 | 93.1 | +0.05 | -0.20 |
| Ribose C5'-C2' | 64.2 | 75.3 | +0.05 | -0.16 |
| Ribose C5'-C3' | 64.3 | 71.7 | +0.11 | +0.04 |
| Deoxyribose C5'-C3' | 68.6 | 78.7 | +0.12 | -0.66 |
| Deoxyribose C5'-C1' | 68.6 | 84.4 | +0.13 | +0.16 |
| Deoxyribose C5'-C4' | 68.6 | 86.6 | +0.15 | -0.08 |
| Ribose C3'-C2' | 71.4 | 75.5 | -0.22 | +0.02 |
| Ribose C3'-C1' | 71.6 | 93.3 | -0.08 | +0.02 |
| Ribose C3'-C4' | 71.7 | 81.8 | +0.03 | -0.13 |
| Ribose C3'-C5' | 72.4 | 64.1 | +0.75 | -0.10 |
| Ribose C2'-C5' | 75.4 | 64.3 | -0.03 | +0.10 |
| Ribose C2'-C3' | 75.5 | 71.3 | +0.06 | -0.35 |
| Ribose C2'-C4' | 75.6 | 82.0 | +0.15 | +0.003 |
| Ribose C2'-C1' | 75.6 | 93.4 | +0.18 | +0.13 |
| Deoxyribose C3'-C4' | 78.9 | 86.5 | -0.49 | -0.28 |
| Deoxyribose C3'-C2' | 79.1 | 39.3 | -0.31 | -0.67 |
| Deoxyribose C3'-C1' | 80.1 | 84.5 | +0.74 | +0.25 |
| Ribose C4'-C1' | 81.8 | 93.1 | -0.12 | -0.20 |
| Ribose C4'-C3' | 82.1 | 71.6 | +0.10 | -0.09 |
| Ribose C4'-C5' | 82.1 | 64.1 | +0.10 | -0.08 |
| Ribose C4'-C2' | 82.3 | 75.5 | +0.31 | +0.06 |
| Deoxyribose C1'-C4' | 83.4 | 87.4 | -0.91 | +0.71 |
| Deoxyribose C1'-C3' | 84.8 | 80.3 | +0.50 | +0.88 |
| Deoxyribose C1'-C5' | 85.0 | 68.3 | +0.75 | -0.16 |
| Deoxyribose C1'-C2' | 85.1 | 39.7 | +0.83 | -0.26 |
| Deoxyribose C4'-C3' | 86.3 | 79.3 | -0.46 | -0.11 |
| Deoxyribose C4'-C2' | 86.6 | 40.9 | -0.13 | +0.90 |
| Deoxyribose C4'-C5' | 86.7 | 68.4 | -0.05 | -0.08 |
| Deoxyribose C4'-C1' | 87.6 | 83.6 | +0.86 | -0.72 |
| Ribose C1'-C5' | 93.2 | 64.0 | -0.08 | -0.18 |
| Ribose C1'-C3' | 93.3 | 71.6 | +0.03 | -0.09 |
| Ribose C1'-C4' | 93.3 | 81.8 | +0.03 | -0.13 |
| Ribose C1'-C2' | 93.5 | 75.2 | +0.25 | -0.27 |

Table S2.

Chemical shift assignments (ω1, ω2) for sugars in the ^13^C-^13^C PDSD spectra of ^13^C, ^15^N-labeled mitochondria, along with their corresponding deviations (δ) calculated by POKY.^[81]^

| ASSIGNMENT OF ^13^C-^13^C PDSD SPECTRA - BASES | | | | |
| --- | --- | --- | --- | --- |
| Correlation | **ω1 (ppm)** | **ω2 (ppm)** | **δ ω1 (ppm)** | **δ ω2 (ppm)** |
| C C5-C6 | 98.1 | 140.2 | -0.06 | -0.15 |
| C C5-C4 | 98.2 | 168.4 | +0.08 | -0.83 |
| U C5-C4 | 102.3 | 170.8 | -1.39 | +0.74 |
| U C5-C6 | 103.3 | 141.6 | -0.36 | -0.11 |
| T C5-C6 | 108.1 | 134.1 | -0.08 | -0.33 |
| T C5-C4 | 108.5 | 165.4 | +0.29 | -1.05 |
| G C5-C4 | 113.0 | 153.5 | -0.08 | -0.03 |
| A C5-C4 | 122.5 | 151.5 | -0.01 | +0.33 |
| A C5-C6 | 122.5 | 162.4 | +0.01 | +0.30 |
| T C6-C5 | 135.4 | 108.0 | +0.91 | -0.21 |
| G C8-C4 | 137.7 | 152.5 | -0.23 | -1.05 |
| A C8-C4 | 137.7 | 151.2 | +0.02 | -0.01 |
| G C8-C6 | 138.3 | 161.7 | +0.36 | +0.04 |
| C C6-C5 | 140.9 | 97.9 | +0.59 | -0.22 |
| C C6-C4 | 141.6 | 169.2 | +1.32 | +0.01 |
| U C6-C5 | 142.8 | 105.1 | +1.07 | +1.41 |
| A C4-C8 | 150.7 | 137.7 | -0.48 | -0.02 |
| A C4-C6 | 151.3 | 161.7 | +0.19 | -0.42 |
| G C4-C5 | 152.5 | 113.2 | -1.03 | +0.08 |
| G C4-C8 | 153.8 | 137.8 | +0.19 | -0.13 |
| G C4-C6 | 154.9 | 161.4 | +1.32 | -0.31 |
| G C6-C4 | 162.0 | 154.1 | +0.27 | +0.57 |
| A C6-C4 | 162.2 | 151.1 | +0.12 | -0.02 |
| T C4-C6 | 167.5 | 133.9 | +1.05 | -0.59 |
| C C4-C5 | 169.1 | 98.3 | -0.12 | +0.21 |
| U C4-C6 | 169.4 | 140.7 | -0.62 | -0.95 |
| U C4-C5 | 169.9 | 104.0 | -0.12 | +0.34 |
| C C4-C6 | 170.1 | 138.6 | +0.94 | -1.75 |

Table S3.

Chemical shift assignments (ω1, ω2) for bases (adenine, cytosine, guanine, thymine, and uracil) in the ^13^C-^13^C PDSD DNP-ssNMR spectra of ^13^C, ^15^N-isotopically labeled mitochondria, along with their corresponding deviations (δ) calculated by POKY.^[81]^

| ASSIGNMENT OF ^13^C-^15^N DCP SPECTRA | | |
| --- | --- | --- |
| Correlation | **^15^N (ppm)** | **^13^C (ppm)** |
| A/G N9-Deoxyribose C1' | 168.3 | 83.9 |
| A/G N9-Ribose C1' | 169.0 | 92.0 |
| C/T N1-Deoxyribose C1' | 143.2 | 84.9 |
| C/U N1-Ribose C1' | 149.0 | 93.2 |
| ASSIGNMENT OF ^1^H-^13^C HETCOR FSLG SPECTRA | | |
| Correlation | **^1^H (ppm)** | **^13^C (ppm)** |
| A H2-C2 | 7.6 | 153.7 |
| C H5-C5 | 6.2 | 97.3 |
| U H5-C5 | 5.5 | 101.8 |
| Deoxyribose H1'-C1' | 6.4 | 84.9 |
| Deoxyribose H2''-C2' | 2.2 | 41.7 |
| Deoxyribose H3'-C3' | 5.3 | 78.4 |
| Deoxyribose H4'-C4' | 4.5 | 86.5 |
| Deoxyribose H5''-C5' | 4.2 | 67.3 |
| Ribose H1'-C1' | 5.8 | 93.2 |
| Ribose H2'-C2' | 4.6 | 75.7 |
| Ribose H3'-C3' | 4.7 | 72.9 |
| Ribose H4'-C4' | 4.8 | 81.5 |
| Ribose H5''-C5' | 4.3 | 63.1 |
| ASSIGNMENT OF ^1^H-^15^N HETCOR FSLG SPECTRA | | |
| Correlation | **^1^H (ppm)** | **^15^N (ppm)** |
| A H(2)6-N6 | 6.7 | 84.3 |
| C H(2)4-N4 | 7.9 | 98.7 |
| G H(2)2-N2 | 6.3 | 75.7 |
| G H1-N1 | 10.2, 13.0 | 149.8 |
| U H3-N3 | 11.4, 13.4, 14.5 | 162.6 |

Table S4.

Chemical shift assignments (^1^H, ^13^C, and ^15^N) for nucleic acids from the ^13^C-^15^N DCP, ^1^H-^13^C, and ^1^H-^15^N HETCOR FSLG DNP-ssNMR spectra of ^13^C, ^15^N-isotopically labeled mitochondria.

Materials and Methods

Mammalian cell culture

HeLa cells (CCL-2^TM^, ATCC, USA) were cultured in Dulbecco’s modified Eagle medium (DMEM, 4.5 g/L D-glucose, Sigma Aldrich, USA) that was supplemented with 10% heat-inactivated fetal bovine serum (FBS) (Gibco, Thermo Fisher Scientific, USA) under a 5% CO_2_ atmosphere at 37 °C. At 80% confluency, the cells were washed with Dulbecco’s phosphate-buffered saline (DPBS) (Sigma-Aldrich, USA), harvested with 0.05% trypsin and 0.02% EDTA 1x DPBS solution (Sigma-Aldrich, USA), and passaged to a new flask supplemented with fresh DMEM containing 10% FBS. ^13^C, ^15^N-labeled mitochondria were grown for 48 hours in BioExpress^®^-6000 Mammalian Cell Growth Media (U-^13^C, 98%; U-^15^N, 98%) supplemented with 10% FBS under 5% CO_2_ atmosphere at 37 °C. Labeled media was prepared according to the manufacturer’s specifications (CIL, USA).

Mitochondrial Isolation

Hela cells were grown to near full confluency and harvested from 6 × T-175 flasks (Sigma-Aldrich) by scrapping after washing once with room temperature (RT) PBS (Sigma-Aldrich). The flasks were washed with 8 mL of PBS and collected into a 50 mL tube (Sigma-Aldrich). Cell counts and viability assessment were performed by trypan blue exclusion staining, and a subset of cells was set aside for analysis by western blot. Cells were pelleted by centrifugation at 300 × g for 5 min at 4 ^o^C. PBS was aspirated, and the pellets was resuspended in 1 mL per 100 mg of wet pellet weight in isotonic buffer (IB+/+) consisting of 220 mM mannitol (Sigma-Aldrich), 70 mM sucrose (Sigma-Aldrich), 5mM HEPES – KOH (pH=7.4), 1 mM EGTA-KOH (pH=7.4), and freshly added 0.5% bovine serum albumin (Sigma-Aldrich) and cOmplete^TM^ protease inhibitor cocktail (Roche, Merck). For one NMR experiment approximately, 1.2 g of cell pellet was harvested. A nitrogen cavitation system was used for cell lysis. The cell pellet was pipetted into the prechilled cell disruption vessel (4639, Parr Instrument Company, USA). The vessel was pressurized by nitrogen to 700 psi and kept in an ice bath for 10 minutes (*46*). The cellular lysate was release from the vessel in a dropwise fashion (1 drop per second) and collected to tube (some lysate stored for later analysis by western blot). The lysate was centrifuged at 600 × g for 10 min at 4 ^o^C. The pellet, containing the nuclear fraction, was resuspended in PBS and stored for analysis by western blot. The supernatant was collected into a fresh tube and centrifuged at 7000 × g for 15 min at 4 ^o^C. A portion of the supernatant was also stored for subsequent analysis by western blot. The mitochondrial pellet was resuspended in 400 µL of IB+/+ buffer and transferred into 4 Eppendorf tubes each containing the Percoll^®^ (Sigma-Alrich) gradient. From top to bottom, the gradient consisted of 0.125 mL of 26% Percoll^®^, 0.375 mL of 52% Percoll^®^, and 0.250 mL of 80% Percoll^®^ – Percoll^®^ solutions were prepared in IB buffer. The tubes were centrifuged at 44000 × g for 45 min at 4 ^o^C. The mitochondrial fraction was located between 26% and 52% and was removed by a needle and syringe. The mitochondria-containing Percoll^®^ solution was diluted 10-fold in chilled IB buffer and centrifuged at 7000 × g for 15 min at 4 ^o^C. The supernatant was discarded, and a pure mitochondrial pellet was obtained. The mitochondrial pellet was resuspended in 300 μL of IB+/+ and prepared for further DNP sample preparation (some pellet was stored for later analysis by western blot).

Ligand treatments onto cells

Hela cells were passaged to 6-well plates supplemented with 3 mL of DMEM containing 10% FBS. At 60% confluency, each well was treated with 0, 1, 5, 10, 15, and 25 µM berberine (Sigma-Aldrich, USA). Microscope pictures (ZOE Fluorescent Cell Imager, Bio-Rad, USA) were taken at 0, 3, 6, 24, 48-hours intervals. Cell division and confluency were analysed by ImageJ software (NIH & LOCI, USA). Individual measurements were done in technical and biological triplicates.

Western Blots

Samples were run in 1× Laemmli buffer down a 1.5 mm tris-glycine gel (4% stacking and 12% resolving) over the course of 2 hours (80 V stacking, 125 V resolving gel). PVDF membranes were submerged into methanol for 5 seconds before blotting for activation. Transfer to PVDF membranes was done by using Bjerrum Schafer-Nielsen buffer (48 mM Tris, 39 mM glycine, 20% methanol, without SDS) and a Bio-Rad semi-dry Trans-Blot turbo transfer system operated at the following settings: 25V, 0.6 A, for 30 min. Membranes submerged in 5% skimmed milk solution (SERVA, Germany) for 30 min at RT. The membranes were subsequently incubated with primary antibodies overnight at 4 ^o^C. The primary antibodies were used at the following dilutions: anti-Actin – 1:2500 (mAb mouse, A3853, Sigma-Aldrich), and anti-VDAC1/Porin 1:500 (mAb mouse 1mg/mL, 14734, Abcam, UK). The membranes were washed 5 times for 10 minutes with 1× TBST buffer (2.4 g Tris, 8.8 g NaCl, pH = 7.6, 0,1% Tween^®^ 20 (Thermo Fisher Scientific, USA)) and treated with 1:3000 secondary antibody goat-anti mouse (IgG HRP, 12-349, Merck) for 1 hour at RT. The membranes were washed 5 times for 10 minutes with 1× TBST buffer and visualized by chemiluminescence on an iBright FL 1500 Imaging system (Thermo Fisher Scientific, USA); machine selected exposure times were utilized. To assess the enrichment of mitochondria in individual cellular fractions following nitrogen cavitation, all lanes were normalized to the actin signal (42 kDa) and compared to mitochondrial protein VDAC1 (31-33 kDa) by iBright Analysis software (Thermo Fisher Scientific, USA).

Isolation of mtRNA

mtRNA was isolated following the mitochondria isolation procedure described above by using a commercial RNeasy kit (Qiagen, The Netherlands). The purity of the isolated mtRNA was assessed on a NanoDrop microvolume spectrophotometer (Thermo Fisher Scientific, USA) based on the Abs_260/280_ ratio. The size of mtRNA fragments was determined by 1.2% agarose gel (Sigma-Aldrich) mixed with GelRed^®^ stain (SCT122, Merck). 6× gel loading purple dye (New England Biolabs, USA) was used for preparing the samples. 1 μg of mtRNA per well was loaded into gel. The gels were visualized using iBright FL 1500 Imaging system (Thermo Fisher Scientific, USA).

Calcium uptake kinetics

The mitochondrial pellet was resuspended in 200 µL of 1× Respiration buffer (RB) (138 mM KCl, 10 mM HEPES-KOH pH = 7.4, 2.5 mM MgCl_2_, 3 mM KH_2_PO_4_-KOH pH = 7.4, 25 µM EDTA) and kept on ice in concentrated conditions. Calcium injection (400 µM CaCl_2_) and RB+/+ working solutions (RB, 5 mM succinate, 5 mM malate, 5 mM glutamate) were prepared directly before fluorescent measurement. The mitochondria sample was diluted to approximately 1 mg/mL and mixed with 2 µL of Calcium Green-5N dye (C3737, Invitrogen, Thermo Fisher Scientific, USA). The sample was incubated and mixed at RT for 5 min. Measurements were conducted on a Cary Eclipse spectrophotometer at 20 °C under constant stirring in kinetics mode with excitation at 503 nm and emission 536 nm, using a high voltage detector, over a time frame 20 minutes. Calcium was injected at 6-minute intervals and repeated twice.^[78]^

Confocal microscopy

Hela cells were passed into cell culture imagining µ-Dish 35 mm (ibidi GmbH, Germany) supplemented by 2 mL of DMEM with 10% FBS 24 hours before the measurement. Twelve hours before the measurement, the cells were exposed to 10 µM berberine; 40 minutes before the measurement, the medium was changed, and DMEM containing the 100 nM MitoTracker^TM^ Red (M7512, Thermo Fisher Scientific, USA) was added. Directly before the measurement, the medium was changed to a fresh DMEM containing 1µg/mL Hoechst 33342 solution (Thermo Fisher Scientific, USA), and the cells were transferred to the microscope for measurement. All microscope images were obtained using a confocal laser scanning microscope Carl Zeiss LSM880 Fast AiryScan AxioObserver 7 SP equipped with alpha Plan-APO 100x/1,46 Oil DIC VIS objective (Zeiss, Germany). The 405 nm, 488 nm, and 561 lasers were used for the excitation with a smart setup mode for emission for individual dyes. Confocal images were processed using ZEN software (Zeiss, Germany).

­

Cryo-EM

The pure isolated mitochondria were diluted to 1.5 mg/mL concentration based on Abs_280_ and 3.5 μL was pipetted onto to a freshly glow discharged Cu200 R2/1 holey carbon grid (Quantifiol^®^, Germany).^[79]^ The gird was subsequently plunge-frozen using a Vitrobot Mark IV (Thermo Fisher Scientific, USA) with blotting paper (595) in a liquid ethane/propane mixture with the following experimental parameters: temperature set to 10 ^o^C, 95% humidity, blot force 0, blotting time 5 s, waiting time 10 s, and drain time set to 0 s. The grid was imaged on a 200 kV Talos Arctica (Thermo Fisher Scientific, USA) equipped with a post-column energy filter and a Gatan K2 detector. The total dose for the cryo-EM image was approximately 10 e^-^/Å^2^. The data were processed using IMOD 4.9 software (University of Colorado, USA).

Preparation of DNP-ssNMR samples

A pellet containing ^13^C, ^15^N-labeled mitochondria isolated from 6× T-175 flasks (~1.4 x 10^8^ cells) using nitrogen cavitation was resuspended in 30 μL of DNP juice (6:4 d_8_-^12^C_3_ glycerol and D_2_O supplemented with 1× IB) and supplemented with 30 mM of SNAPol-1.^[51]^ The sample was pipetted into a 3.2 mm sapphire DNP rotor in 10 μL aliquots and centrifuged at 7 000 × g for 3 min at 4 ^o^C until only the pellet remained. Excess DNP juice was removed with a paper towel after each centrifugation step. After the last round of centrifugation, the rotor was closed, the bottom was marked for MAS detection, and it was immersed in liquid nitrogen. For the DNP sample with berberine, mitochondria isolated after treatment were incubated with 30 µM berberine for 20 minutes, washed with fresh 1× IB, and then mixed with DNP juice. ^13^C, ^15^N-labeled mtRNA was isolated from DNP mitochondrial sample after ssNMR measurement. Isolated mtRNA was lyophilized overnight and resuspended in 30 μL of DNP juice (6:4 d_8_-^12^C_3_ glycerol and D_2_O supplemented with 1× IB) supplemented with 30 mM of SNAPol-1. The rotor was prepared for DNP-ssNMR as described above.

DNP-enhanced MAS ssNMR spectroscopy

DNP-ssNMR data were acquired at a static magnetic field strength of 18.8 T (Bruker Corporation, USA), corresponding to a ^1^H Larmor frequency of 800 MHz. The spectrometer was equipped with an AVANCE NEO console (Bruker Corporation, USA), a 3.2 mm HXY low-temperature DNP probe, permanent sweep coil, and a 9.7 T - 527 GHZ gyrotron. Measurements were conducted at a MAS rate of either 8 or 10 kHz at 100K. Spectra requiring proton-decoupling utilized SPINAL-64^[80]^ at a strength of 92 kHz. Data were collected at 60 mA of microwave irradiation. Data were processed by Topspin 4.4.0 (Bruker), Poky,^[81]^ and MNova v15.0.1 (Mestrelab Research, Spain) software. Chemical shift assignments were obtained from the BMRB and literature sources.^[41, 47, 74, 82]^

*^13^C /^15^N enhancement and polarization buildup:* ^13^C-enhancement was determined indirectly by utilizing a ^1^H-^13^C adiabatic cross polarization (CP) with an asymmetric 70 μs contact time, 2 s recycle delay, 500 ppm spectral window (1458 points) and an 8 step-phase cycle. CP spin locking was optimized for each sample individually but hovered around 65 kHz for ^13^C and 73 for kHz ^1^H respectively. Relaxation measurements were obtained via a saturation recovery adiabatic ^1^H-^13^C CP. DNP on/off spectra were acquired with 2048 scans whereas buildup was acquired with 32 scans per delay. 1D spectra were processed in Topspin 4.1.0 with line broadening (150 Hz) being applied prior to Fourier transformation (FT). The ^15^N-enhancment was determined exactly as discussed above. The carrier frequency was placed at 120 ppm. A CP spin lock of 50 kHz was used for ^15^N. Spectra were recorded with a spectral window of 1000 ppm (2048 points) and 2048 scans for the DNP-ON spectra and 8192 scans for the DNP-OFF spectra. T_B_ was determined indirectly through ^1^H-^13^C saturation recovery; resulting curves were fit with up to 3 exponentials in Prism 9.^[52]^

*^13^C-^13^C PDSD:* PDSD experiments were acquired with a 30 ms spin diffusion time and MAS of 10 kHz. The carrier frequency was placed at 80 ppm. All PDSD spectra were recorded 328 scans and with a spectral window of 407 ppm (2368 points) in the direct and 261 ppm (328 points) in the indirect dimension.

*^13^C, ^15^N DCP:* Magnetization transfer from ^1^H to ^15^N utilized a 310 μs contact time with the following spin lock powers: 60 kHz for ^1^H and 48 kHz for ^15^N.^[63]^ Magnetization transfer from ^15^N to ^13^C used a contact time of 3.7 ms with the following spin lock powers: 48 kHz for ^15^N and 17 kHz for ^13^C. All DCP spectra were recorded with 768 scans and with a spectral window of 407 ppm (2368 points) in the direct and 650 ppm (328 points) in the indirect dimension. All DCP spectra were acquired at 8 kHz MAS.

*^1^H-^13^C and ^1^H-^15^N FSLG HETCOR:* A proton nutation frequency of 100 kHz was used, and the proton offset during Lee-Goldburg evolution was set to 2 kHz.^[61]^ To probe single bond connections a ^1^H-^13^C/^15^N CP contact pulse width of 100 μs was used. Contact times were increased to 400 μs to detect longer connections.^[33]^ Both­ ^13^C detected FSLG-HETCOR spectra were recorded with 464 scans and a spectral window of 407 ppm (2380 points) in the direct and 40 ppm (128 points) in the indirect dimension. Both ^15^N detected FSLG were recorded with 1024 scans and a spectral window of 455 ppm (2380 points) in the direct and 40 ppm (64 points) in the indirect dimension. Both ^13^C and ^15^N detected spectra were acquired with at 10 kHz MAS.
